
JOURNAL INFORMATION
==============================
NLM Title Abbreviation: Can Med Educ J
Journal ID: Can Med Educ J
NLM Journal ID: 101560935
Journal ID: CMEJ

Canadian Medical Education Journal

EISSN: 1923-1202
Publisher: University of Saskatchewan

ARTICLE INFORMATION
==============================
PMCID: PMC6448503
Article ID: CMEJ-10-e029
Article version: 1
Subjects: Abstracts

Oral Abstracts

Electronic publication date: 2019 Apr 3
Publication date: 2019 Apr
Volume: 10
Issue: 2
First page: e029
Last page: e197
Copyright year: 2019
License: This is an Open Journal Systems article distributed under the terms of the Creative Commons Attribution License (http://creativecommons.org/licenses/by/2.0) which permits unrestricted use, distribution, and reproduction in any medium, provided the original work is properly cited.
License URL: https://creativecommons.org/licenses/by/2.0/

==============================
JOURNAL INFORMATION
==============================
NLM Title Abbreviation: Can Med Educ J
Journal ID: Can Med Educ J
NLM Journal ID: 101560935
Journal ID: CMEJ

Canadian Medical Education Journal

EISSN: 1923-1202
Publisher: University of Saskatchewan

ARTICLE INFORMATION
==============================
Subjects: Oa - 1 – 1

Breaking down the barriers to mistreatment reporting: the University of Calgary mistreatment plan

Paget Mike University of Calgary

Heyns Marguerite University of Calgary

Doig Christopher University of Calgary

Woloschuk Wayne University of Calgary

de Groot Janet University of Calgary

Jenkins Deirdre University of Calgary

Busche Kevin University of Calgary

Coderre Sylvain University of Calgary

Electronic publication date: 2019 Apr 3

JOURNAL INFORMATION
==============================
NLM Title Abbreviation: Can Med Educ J
Journal ID: Can Med Educ J
NLM Journal ID: 101560935
Journal ID: CMEJ

Canadian Medical Education Journal

EISSN: 1923-1202
Publisher: University of Saskatchewan

ARTICLE INFORMATION
==============================
Subjects: Oa - 1 – 2

A national look at the influence of accreditation on Medical Council of Canada Qualifying Examination (MCCQE Part I) scores

Wood Timothy University of Ottawa

Roy Marguerite Medical Council of Canada

Eva Kevin University of British Columbia

Blouin Danielle Queen’s University

Electronic publication date: 2019 Apr 3

JOURNAL INFORMATION
==============================
NLM Title Abbreviation: Can Med Educ J
Journal ID: Can Med Educ J
NLM Journal ID: 101560935
Journal ID: CMEJ

Canadian Medical Education Journal

EISSN: 1923-1202
Publisher: University of Saskatchewan

ARTICLE INFORMATION
==============================
Subjects: Oa - 1 – 3

Accreditation and Canadian medical schools: Harnessing the power of affinity mapping for CQI

Venance Shannon CACMS/CACME

Blouin Danielle CACMS/CACME

Electronic publication date: 2019 Apr 3

JOURNAL INFORMATION
==============================
NLM Title Abbreviation: Can Med Educ J
Journal ID: Can Med Educ J
NLM Journal ID: 101560935
Journal ID: CMEJ

Canadian Medical Education Journal

EISSN: 1923-1202
Publisher: University of Saskatchewan

ARTICLE INFORMATION
==============================
Subjects: Oa - 1 – 4

A National Survey of Canadian Program Directors on their Perceptions of Accreditation and CanMEDS Implementation

Dore Kelly McMaster University

Bogie Bryce McMaster University

Finlay Karen McMaster University

Saperson Karen McMaster University

Wasi Parveen McMaster University

Electronic publication date: 2019 Apr 3

JOURNAL INFORMATION
==============================
NLM Title Abbreviation: Can Med Educ J
Journal ID: Can Med Educ J
NLM Journal ID: 101560935
Journal ID: CMEJ

Canadian Medical Education Journal

EISSN: 1923-1202
Publisher: University of Saskatchewan

ARTICLE INFORMATION
==============================
Subjects: Oa - 1 – 5

PSP Module Evolution Project: Leading CPD & Quality Improvement Practice

Tajani Sarah University of British Columbia

Beamish Laura University of British Columbia

Crew Mallory Practice Support Program, Doctors of BC

Hobson Bruce University of British Columbia

Lam Vivian University of British Columbia

Sze Shirley University of British Columbia

Electronic publication date: 2019 Apr 3

JOURNAL INFORMATION
==============================
NLM Title Abbreviation: Can Med Educ J
Journal ID: Can Med Educ J
NLM Journal ID: 101560935
Journal ID: CMEJ

Canadian Medical Education Journal

EISSN: 1923-1202
Publisher: University of Saskatchewan

ARTICLE INFORMATION
==============================
Subjects: Oa - 1 – 6

Improving Resident Education on Practice Management

Neferu Ramona McMaster University

Bhatia Meghan University of Toronto

Chu Laura Memorial – University of Newfoundland

Doshi Samik University of Toronto

Lerner Jordyn University of Manitoba

Tang Brandon University of British Columbia

Electronic publication date: 2019 Apr 3

JOURNAL INFORMATION
==============================
NLM Title Abbreviation: Can Med Educ J
Journal ID: Can Med Educ J
NLM Journal ID: 101560935
Journal ID: CMEJ

Canadian Medical Education Journal

EISSN: 1923-1202
Publisher: University of Saskatchewan

ARTICLE INFORMATION
==============================
Subjects: Oa - 2 – 1

The Modified Personal Interview's Predictive and Consequential Validity: Can the Compromise Approach Work?

(Mahan) Kulasegaram Kulamakan University of Toronto

Latter David University of Toronto

Hanson Mark University of Toronto

Electronic publication date: 2019 Apr 3

JOURNAL INFORMATION
==============================
NLM Title Abbreviation: Can Med Educ J
Journal ID: Can Med Educ J
NLM Journal ID: 101560935
Journal ID: CMEJ

Canadian Medical Education Journal

EISSN: 1923-1202
Publisher: University of Saskatchewan

ARTICLE INFORMATION
==============================
Subjects: Oa - 2 – 2

Contrasting Patient and Standardized Patient Engagement in Medical Student and Resident Selection

D. Hanson Mark University of Toronto

Pang Celeste University of Toronto

McLeod Justine SickKids

Springall Elena University of Toronto

Kulasegaram Kulamakan University of Toronto

Eva Kevin University of British Columbia

Electronic publication date: 2019 Apr 3

JOURNAL INFORMATION
==============================
NLM Title Abbreviation: Can Med Educ J
Journal ID: Can Med Educ J
NLM Journal ID: 101560935
Journal ID: CMEJ

Canadian Medical Education Journal

EISSN: 1923-1202
Publisher: University of Saskatchewan

ARTICLE INFORMATION
==============================
Subjects: Oa - 2 – 3

A Diversity Success Story: A Tailored Application Stream Leads to Increase of Black Students in Medical School

Lee Hana University of Toronto

Latter David University of Toronto

Jackowetz Lindsay University of Toronto

Taylor Leslie University of Toronto

Okafor Ike University of Toronto

Robinson Lisa University of Toronto

Nnorom Onyenyechukwu University of Toronto

Electronic publication date: 2019 Apr 3

JOURNAL INFORMATION
==============================
NLM Title Abbreviation: Can Med Educ J
Journal ID: Can Med Educ J
NLM Journal ID: 101560935
Journal ID: CMEJ

Canadian Medical Education Journal

EISSN: 1923-1202
Publisher: University of Saskatchewan

ARTICLE INFORMATION
==============================
Subjects: Oa - 2 – 4

"If you re-lived your life, would you still want to become a physician?": Unexpected Patterns of Ambivalence from Incoming MD Students through to Exiting Residents

Bandiera Glen University of Toronto

Babcock Glenys University of Toronto

Ruetalo Mariela University of Toronto

Latter David University of Toronto

Lee Hana University of Toronto

Robinson Lisa University of Toronto

Electronic publication date: 2019 Apr 3

JOURNAL INFORMATION
==============================
NLM Title Abbreviation: Can Med Educ J
Journal ID: Can Med Educ J
NLM Journal ID: 101560935
Journal ID: CMEJ

Canadian Medical Education Journal

EISSN: 1923-1202
Publisher: University of Saskatchewan

ARTICLE INFORMATION
==============================
Subjects: Oa - 2 – 5

Novel approach to standard setting an admission panel interview

Barber Cassandra Western University

Hammond Robert Western University

Joy Tisha Western University

Chahine Saad Western University

Electronic publication date: 2019 Apr 3

JOURNAL INFORMATION
==============================
NLM Title Abbreviation: Can Med Educ J
Journal ID: Can Med Educ J
NLM Journal ID: 101560935
Journal ID: CMEJ

Canadian Medical Education Journal

EISSN: 1923-1202
Publisher: University of Saskatchewan

ARTICLE INFORMATION
==============================
Subjects: Oa - 2 – 6

How to Integrate Situational Judgement Test Scores into Existing Medical Admissions Processes: A Simulation Study

Juster Fern New York Medical College

Zou Christopher Altus Assessments

Reiter Harold McMaster University

Dore Kelly McMaster University

Electronic publication date: 2019 Apr 3

JOURNAL INFORMATION
==============================
NLM Title Abbreviation: Can Med Educ J
Journal ID: Can Med Educ J
NLM Journal ID: 101560935
Journal ID: CMEJ

Canadian Medical Education Journal

EISSN: 1923-1202
Publisher: University of Saskatchewan

ARTICLE INFORMATION
==============================
Subjects: Oa - 3 – 1

The Sociomaterial Implications of Procedural Variation for Assessment in Competency-Based Medical Education

Ott Mary Western University

Cristancho Sayra Western University

Apramian Tavis Western University

Lingard Lorelei Western University

Roth Kathryn Western University

Electronic publication date: 2019 Apr 3

JOURNAL INFORMATION
==============================
NLM Title Abbreviation: Can Med Educ J
Journal ID: Can Med Educ J
NLM Journal ID: 101560935
Journal ID: CMEJ

Canadian Medical Education Journal

EISSN: 1923-1202
Publisher: University of Saskatchewan

ARTICLE INFORMATION
==============================
Subjects: Oa - 3 – 2

Conflict Management: Perspectives from a Chief Resident Workshop

Desanghere Loni University of Saskatchewan

Saxena Anurag University of Saskatchewan

Rohr Betty University of Saskatchewan

Electronic publication date: 2019 Apr 3

JOURNAL INFORMATION
==============================
NLM Title Abbreviation: Can Med Educ J
Journal ID: Can Med Educ J
NLM Journal ID: 101560935
Journal ID: CMEJ

Canadian Medical Education Journal

EISSN: 1923-1202
Publisher: University of Saskatchewan

ARTICLE INFORMATION
==============================
Subjects: Oa - 3 – 3

Application rates to surgical residency programs in Canada

Dow Todd Dalhousie University

McGuire Connor Dalhousie University

Crawley Emma Dalhousie University

Davies Dafydd Dalhousie University

Electronic publication date: 2019 Apr 3

JOURNAL INFORMATION
==============================
NLM Title Abbreviation: Can Med Educ J
Journal ID: Can Med Educ J
NLM Journal ID: 101560935
Journal ID: CMEJ

Canadian Medical Education Journal

EISSN: 1923-1202
Publisher: University of Saskatchewan

ARTICLE INFORMATION
==============================
Subjects: Oa - 3 – 4

Developing a medical assistance in dying curriculum in a family medicine residency training program

MacDonald Susan Queen’s University

LeBlanc Sarah Queen’s University

Dalgarno Nancy Queen’s University

Zimmerman Daniel Queen’s University

Schultz Karen Queen’s University

Johnston Emily Calgary West Central Primary Care Network

Martin Mary Queen’s University

Electronic publication date: 2019 Apr 3

JOURNAL INFORMATION
==============================
NLM Title Abbreviation: Can Med Educ J
Journal ID: Can Med Educ J
NLM Journal ID: 101560935
Journal ID: CMEJ

Canadian Medical Education Journal

EISSN: 1923-1202
Publisher: University of Saskatchewan

ARTICLE INFORMATION
==============================
Subjects: Oa - 3 – 5

Working in the dead of night: Exploring the transition to after-hours duty

Walzak Ali University of British Columbia

Butler Deborah University of British Columbia

Bates Joanna University of British Columbia

Farrell Laura University of British Columbia

Law Bosco McMaster University

Pratt Daniel University of British Columbia

Electronic publication date: 2019 Apr 3

JOURNAL INFORMATION
==============================
NLM Title Abbreviation: Can Med Educ J
Journal ID: Can Med Educ J
NLM Journal ID: 101560935
Journal ID: CMEJ

Canadian Medical Education Journal

EISSN: 1923-1202
Publisher: University of Saskatchewan

ARTICLE INFORMATION
==============================
Subjects: Oa - 3 – 6

Are residents paying attention to your lecture? Neural signatures of mind wandering in orthopaedic surgery residents during live lectures

Acai Anita McMaster University

Dhindsa Kiret McMaster University

Wagner Natalie McMaster University

Bosynak Dan McMaster University

Kelly Stephen McMaster University

Bhandari Mohit McMaster University

Petrisor Bradley McMaster University

R. Sonnadara Ranil McMaster University

Electronic publication date: 2019 Apr 3

JOURNAL INFORMATION
==============================
NLM Title Abbreviation: Can Med Educ J
Journal ID: Can Med Educ J
NLM Journal ID: 101560935
Journal ID: CMEJ

Canadian Medical Education Journal

EISSN: 1923-1202
Publisher: University of Saskatchewan

ARTICLE INFORMATION
==============================
Subjects: Oa - 4 – 1

Clarifying and contextualizing the relationship between evidence-based practice and clinical reasoning in rehabilitation education

Thomas Aliki McGill

Young Meredith McGill

Yeung Euson University of Toronto

Lubarsky Stuart McGill

Dory Valerie McGill

Varpio Lara Uniformed Services University of the Health Sciences, McGill University

Macdonald Mary-Ellen McGill

Electronic publication date: 2019 Apr 3

JOURNAL INFORMATION
==============================
NLM Title Abbreviation: Can Med Educ J
Journal ID: Can Med Educ J
NLM Journal ID: 101560935
Journal ID: CMEJ

Canadian Medical Education Journal

EISSN: 1923-1202
Publisher: University of Saskatchewan

ARTICLE INFORMATION
==============================
Subjects: Oa - 4 – 2

Implementing self-explanation and structured reflection to support clinical reasoning in undergraduate medical students: a longitudinal study

Chamberland Martine Université de Sherbrooke

Setrakian Jean Université de Sherbrooke

Bergeron Linda Université de Sherbrooke

St-Onge Christina Université de Sherbrooke

Plaisance Martin Université de Sherbrooke

Varpio Lara Uniformed Services University of the Health Sciences

Thomas Aliki McGill

Electronic publication date: 2019 Apr 3

JOURNAL INFORMATION
==============================
NLM Title Abbreviation: Can Med Educ J
Journal ID: Can Med Educ J
NLM Journal ID: 101560935
Journal ID: CMEJ

Canadian Medical Education Journal

EISSN: 1923-1202
Publisher: University of Saskatchewan

ARTICLE INFORMATION
==============================
Subjects: Oa - 4 – 3

A scoping study of the role of ambiguity, uncertainty, and complexity in clinical reasoning

Young Meredith McGill

Thomas Aliki McGill

Lubarsky Stuart McGill

Dory Valerie McGill

Torabi Nazi University Health Network, University of Toronto

Bhanji Farhan The Royal College of Physicians and Surgeons

Durning Steven Uniformed Services University of the Health Professions

Electronic publication date: 2019 Apr 3

JOURNAL INFORMATION
==============================
NLM Title Abbreviation: Can Med Educ J
Journal ID: Can Med Educ J
NLM Journal ID: 101560935
Journal ID: CMEJ

Canadian Medical Education Journal

EISSN: 1923-1202
Publisher: University of Saskatchewan

ARTICLE INFORMATION
==============================
Subjects: Oa - 4 – 4

Developing Interviewing and Clinical Reasoning Skills with a Novel Low-Cost Virtual Patient Simulator

Kaminska Malgorzata University of Northern British Columbia

Franke Richard Athabasca University,

Electronic publication date: 2019 Apr 3

JOURNAL INFORMATION
==============================
NLM Title Abbreviation: Can Med Educ J
Journal ID: Can Med Educ J
NLM Journal ID: 101560935
Journal ID: CMEJ

Canadian Medical Education Journal

EISSN: 1923-1202
Publisher: University of Saskatchewan

ARTICLE INFORMATION
==============================
Subjects: Oa - 4 – 5

Early development of clinical skills in a competence based undergraduate medical program

Plaisance Martin Université de Sherbrooke

Germain Isabelle Université de Sherbrooke

Mathieu Sylvie Université de Sherbrooke

Houde Ghislaine Université de Sherbrooke

Electronic publication date: 2019 Apr 3

JOURNAL INFORMATION
==============================
NLM Title Abbreviation: Can Med Educ J
Journal ID: Can Med Educ J
NLM Journal ID: 101560935
Journal ID: CMEJ

Canadian Medical Education Journal

EISSN: 1923-1202
Publisher: University of Saskatchewan

ARTICLE INFORMATION
==============================
Subjects: Oa - 4 – 6

"You cut like a girl" - A sequential explanatory mixed methods study of gender-based discrimination among surgical residents

Bonneville Gabrielle University of Calgary

Brown Allison University of Calgary

Glaze Sarah University of Calgary

Electronic publication date: 2019 Apr 3

JOURNAL INFORMATION
==============================
NLM Title Abbreviation: Can Med Educ J
Journal ID: Can Med Educ J
NLM Journal ID: 101560935
Journal ID: CMEJ

Canadian Medical Education Journal

EISSN: 1923-1202
Publisher: University of Saskatchewan

ARTICLE INFORMATION
==============================
Subjects: Oa - 5 – 1

Customizing Support for New-to-Rural-Emergency Physicians through Peer Clinical Coaching

Thomson Claire University of British Columbia

Bluman Bob University of British Columbia

Leung Dilys University of British Columbia

Overhill Kirstie University of British Columbia

Electronic publication date: 2019 Apr 3

JOURNAL INFORMATION
==============================
NLM Title Abbreviation: Can Med Educ J
Journal ID: Can Med Educ J
NLM Journal ID: 101560935
Journal ID: CMEJ

Canadian Medical Education Journal

EISSN: 1923-1202
Publisher: University of Saskatchewan

ARTICLE INFORMATION
==============================
Subjects: Oa - 5 – 2

Reflection and Professional Identity: Implementation of a Longitudinal Course at the Undergraduate Medical Education Level

Desilets Valerie Université de Sherbrooke

Graillon Ann Université de Sherbrooke

Xhignesse Marianne Université de Sherbrooke

Ouellet Kathleen Université de Sherbrooke

St-Onge Christina Université de Sherbrooke

Electronic publication date: 2019 Apr 3

JOURNAL INFORMATION
==============================
NLM Title Abbreviation: Can Med Educ J
Journal ID: Can Med Educ J
NLM Journal ID: 101560935
Journal ID: CMEJ

Canadian Medical Education Journal

EISSN: 1923-1202
Publisher: University of Saskatchewan

ARTICLE INFORMATION
==============================
Subjects: Oa - 5 – 3

Improving role modeling in clinical educators

Mortaz Hejri Sara Tehran University Of Medical Sciences

Mohammadi Elaheh Tehran University Of Medical Sciences

Shahsavari Hooman Tehran University Of Medical Sciences

Sohrabpour Amirali Tehran University Of Medical Sciences

Mirzazadeh Azim Tehran University Of Medical Sciences

Electronic publication date: 2019 Apr 3

JOURNAL INFORMATION
==============================
NLM Title Abbreviation: Can Med Educ J
Journal ID: Can Med Educ J
NLM Journal ID: 101560935
Journal ID: CMEJ

Canadian Medical Education Journal

EISSN: 1923-1202
Publisher: University of Saskatchewan

ARTICLE INFORMATION
==============================
Subjects: Oa - 5 – 4

"Intro To Code Blue" Curriculum: Using OSCE-like Learning Checklists in 4 Tandem, Coached, Low-Fidelity Simulations to Consolidate Medical Students' Approach to Acute Care Situations

Seto Anthony University of Calgary

Electronic publication date: 2019 Apr 3

JOURNAL INFORMATION
==============================
NLM Title Abbreviation: Can Med Educ J
Journal ID: Can Med Educ J
NLM Journal ID: 101560935
Journal ID: CMEJ

Canadian Medical Education Journal

EISSN: 1923-1202
Publisher: University of Saskatchewan

ARTICLE INFORMATION
==============================
Subjects: Oa - 5 – 6

Clinical Faculty Mentoring: A pilot Program in Academic Medicine

Ameyaw Stephanie University of British Columbia

Lynn Brenna University of British Columbia

Parhar Gurdeep University of British Columbia

Paul Susan University of British Columbia

Electronic publication date: 2019 Apr 3

JOURNAL INFORMATION
==============================
NLM Title Abbreviation: Can Med Educ J
Journal ID: Can Med Educ J
NLM Journal ID: 101560935
Journal ID: CMEJ

Canadian Medical Education Journal

EISSN: 1923-1202
Publisher: University of Saskatchewan

ARTICLE INFORMATION
==============================
Subjects: Oa - 6 – 1

Conceptualization of Competency-Based Medical Education, Competency, and Competence within the CPD/CME and Residency Family Medicine Educational Literature

Lochnan Heather University of Ottawa

Hendry Paul University of Ottawa

Kitto Simon University of Ottawa

Walsh Allyn University of Ottawa

Viner Gary University of Ottawa

Oandasan Ivy University of Toronto

Electronic publication date: 2019 Apr 3

JOURNAL INFORMATION
==============================
NLM Title Abbreviation: Can Med Educ J
Journal ID: Can Med Educ J
NLM Journal ID: 101560935
Journal ID: CMEJ

Canadian Medical Education Journal

EISSN: 1923-1202
Publisher: University of Saskatchewan

ARTICLE INFORMATION
==============================
Subjects: Oa - 6 – 2

Innovative multimodal program training ultrasound experts in continuing medical education

Ouellet Annie Université de Sherbrooke

Gosselin Émilie Université de Sherbrooke

Bertholet Catherine Université de Sherbrooke

Mathieu Luc Université de Sherbrooke

Electronic publication date: 2019 Apr 3

JOURNAL INFORMATION
==============================
NLM Title Abbreviation: Can Med Educ J
Journal ID: Can Med Educ J
NLM Journal ID: 101560935
Journal ID: CMEJ

Canadian Medical Education Journal

EISSN: 1923-1202
Publisher: University of Saskatchewan

ARTICLE INFORMATION
==============================
Subjects: Oa - 6 – 3

From lit to lightbulbs: A comprehensive approach to program and event needs assessments

van Wylick Richard Queen’s University

Soleas Eleftherios Queen’s University

Dalgarno Nancy Queen’s University

Harle Ingrid Queen’s University

De sousa Mikaila Queen’s University

Electronic publication date: 2019 Apr 3

JOURNAL INFORMATION
==============================
NLM Title Abbreviation: Can Med Educ J
Journal ID: Can Med Educ J
NLM Journal ID: 101560935
Journal ID: CMEJ

Canadian Medical Education Journal

EISSN: 1923-1202
Publisher: University of Saskatchewan

ARTICLE INFORMATION
==============================
Subjects: Oa - 6 – 4

Charting a path to scholarship in Continuing Professional Development

Paton Morag University of Toronto

Rowland Paula University of Toronto

Tavares Walter University of Toronto

Schneeweiss Suzan University of Toronto

Ginsburg Shiphra University of Toronto

Electronic publication date: 2019 Apr 3

JOURNAL INFORMATION
==============================
NLM Title Abbreviation: Can Med Educ J
Journal ID: Can Med Educ J
NLM Journal ID: 101560935
Journal ID: CMEJ

Canadian Medical Education Journal

EISSN: 1923-1202
Publisher: University of Saskatchewan

ARTICLE INFORMATION
==============================
Subjects: Oa - 6 – 5

Exploring Potential Mechanisms of Learning in Project ECHO: Understanding The "Why"

Sockalingam Sanjeev University of Toronto

Zhou Carrol University of Toronto

Rajaratnam Thiyake Centre for Addiction and Mental Health

Serhal Eva Centre for Addiction and Mental Health

Crawford Allison University of Toronto

Mylopoulos Maria University of Toronto

Electronic publication date: 2019 Apr 3

JOURNAL INFORMATION
==============================
NLM Title Abbreviation: Can Med Educ J
Journal ID: Can Med Educ J
NLM Journal ID: 101560935
Journal ID: CMEJ

Canadian Medical Education Journal

EISSN: 1923-1202
Publisher: University of Saskatchewan

ARTICLE INFORMATION
==============================
Subjects: Oa - 6 – 6

Improving patients' outcomes through better screening, treatment and follow up of lung cancer

Wade Patricia Fédération des médecins spécialistes du Québec

J. Daniel Sam Fédération des médecins spécialistes du Québec

Merlos Beatriz Fédération des médecins spécialistes du Québec

Tremblay Martin Fédération des médecins spécialistes du Québec

Electronic publication date: 2019 Apr 3

JOURNAL INFORMATION
==============================
NLM Title Abbreviation: Can Med Educ J
Journal ID: Can Med Educ J
NLM Journal ID: 101560935
Journal ID: CMEJ

Canadian Medical Education Journal

EISSN: 1923-1202
Publisher: University of Saskatchewan

ARTICLE INFORMATION
==============================
Subjects: Oa - 7 – 1

Retention of visa trainees following post-graduate medical residency training in Canada.

Mathews Maria Western University

Bourgeault Ivy University of Ottawa

Yi Yanqing Memorial – University of Newfoundland

Koudieh Dania The Association of Faculties of Medicine of Canada

Barer Morris University of British Columbia

Hedden Lindsay University of British Columbia

Marshall Emily Dalhousie University

Electronic publication date: 2019 Apr 3

JOURNAL INFORMATION
==============================
NLM Title Abbreviation: Can Med Educ J
Journal ID: Can Med Educ J
NLM Journal ID: 101560935
Journal ID: CMEJ

Canadian Medical Education Journal

EISSN: 1923-1202
Publisher: University of Saskatchewan

ARTICLE INFORMATION
==============================
Subjects: Oa - 7 – 2

Design of a Disease Based Clinical Training Curriculum in Paediatric Haematology/Oncology: Are we preparing our trainees better for independent practice?

Cada Michaela University of Toronto

Punnett Angela University of Toronto

Wilejto Marta Western University

Electronic publication date: 2019 Apr 3

JOURNAL INFORMATION
==============================
NLM Title Abbreviation: Can Med Educ J
Journal ID: Can Med Educ J
NLM Journal ID: 101560935
Journal ID: CMEJ

Canadian Medical Education Journal

EISSN: 1923-1202
Publisher: University of Saskatchewan

ARTICLE INFORMATION
==============================
Subjects: Oa - 7 – 3

Easing the Transition to Residency: An Internal Medicine Boot Camp Pilot

Goodliffe Laura McMaster University

Merali Zahra McMaster University

Brandt Vegas Daniel McMaster University

Martin Leslie McMaster University

Electronic publication date: 2019 Apr 3

JOURNAL INFORMATION
==============================
NLM Title Abbreviation: Can Med Educ J
Journal ID: Can Med Educ J
NLM Journal ID: 101560935
Journal ID: CMEJ

Canadian Medical Education Journal

EISSN: 1923-1202
Publisher: University of Saskatchewan

ARTICLE INFORMATION
==============================
Subjects: Oa - 7 – 4

Learner Handover: How does it influence assessment?

Shaw Tammy University of Ottawa

Pugh Debra University of Ottawa

Touchie Claire University of Ottawa

J. Wood Timothy University of Ottawa

Humphrey-Murto Susan University of Ottawa

Electronic publication date: 2019 Apr 3

JOURNAL INFORMATION
==============================
NLM Title Abbreviation: Can Med Educ J
Journal ID: Can Med Educ J
NLM Journal ID: 101560935
Journal ID: CMEJ

Canadian Medical Education Journal

EISSN: 1923-1202
Publisher: University of Saskatchewan

ARTICLE INFORMATION
==============================
Subjects: Oa - 7 – 5

The influence of prior performance information on ratings of present performance: Implications for learner handover: A Scoping Review

Humphrey-Murto Susan University of Ottawa

LeBlanc Aaron University of Ottawa

Touchie Claire Medical Council of Canada

Debra Pugh University of Ottawa

J. Wood Timothy University of Ottawa

Cowley Lindsday University of Ottawa

Shaw Tammy University of Ottawa

Electronic publication date: 2019 Apr 3

JOURNAL INFORMATION
==============================
NLM Title Abbreviation: Can Med Educ J
Journal ID: Can Med Educ J
NLM Journal ID: 101560935
Journal ID: CMEJ

Canadian Medical Education Journal

EISSN: 1923-1202
Publisher: University of Saskatchewan

ARTICLE INFORMATION
==============================
Subjects: Oa - 7 – 6

A multi-year pilot study of a longitudinal mindfulness curriculum in undergraduate medical education

MacLean Heather University of Ottawa

Braschi Emelie University of Ottawa

Archibald Douglas University of Ottawa

Sanchez-Campos Millaray University of Ottawa

Jebanesan Danusha University of Ottawa

Koszycki Diana University of Ottawa

Gonsalves Carol University of Ottawa

Electronic publication date: 2019 Apr 3

JOURNAL INFORMATION
==============================
NLM Title Abbreviation: Can Med Educ J
Journal ID: Can Med Educ J
NLM Journal ID: 101560935
Journal ID: CMEJ

Canadian Medical Education Journal

EISSN: 1923-1202
Publisher: University of Saskatchewan

ARTICLE INFORMATION
==============================
Subjects: Oa - 8 – 1

Indigenous students continue to face racial adversity at Canadian medical schools

Young Brent Dalhousie University

Sauve Amanda University of Toronto

Bombay Amy Dalhousie University

Electronic publication date: 2019 Apr 3

JOURNAL INFORMATION
==============================
NLM Title Abbreviation: Can Med Educ J
Journal ID: Can Med Educ J
NLM Journal ID: 101560935
Journal ID: CMEJ

Canadian Medical Education Journal

EISSN: 1923-1202
Publisher: University of Saskatchewan

ARTICLE INFORMATION
==============================
Subjects: Oa - 8 – 2

Against the odds: Experiences of Canadian Medical Learners with Disabilities

Liao Pamela University of Toronto

Jarus Tal University of British Columbia

Das Shyama University of British Columbia

MD Krejcik Vera University of British Columbia

Tikhonova Julia University of British Columbia

Battalova Alfiya University of British Columbia

Electronic publication date: 2019 Apr 3

JOURNAL INFORMATION
==============================
NLM Title Abbreviation: Can Med Educ J
Journal ID: Can Med Educ J
NLM Journal ID: 101560935
Journal ID: CMEJ

Canadian Medical Education Journal

EISSN: 1923-1202
Publisher: University of Saskatchewan

ARTICLE INFORMATION
==============================
Subjects: Oa - 8 – 3

Identity, Trust, and Care: Moving through discomfort towards meaningful care for transgender patients

Baker Lindsay University of Toronto

Boyd Victoria University of Toronto

Kangasjarvi Emilia University of Toronto

Ng Stella University of Toronto

McNeil Beck The 519 Church Street Community Centre

Electronic publication date: 2019 Apr 3

JOURNAL INFORMATION
==============================
NLM Title Abbreviation: Can Med Educ J
Journal ID: Can Med Educ J
NLM Journal ID: 101560935
Journal ID: CMEJ

Canadian Medical Education Journal

EISSN: 1923-1202
Publisher: University of Saskatchewan

ARTICLE INFORMATION
==============================
Subjects: Oa - 8 – 4

The Implicit Association Test in Health Professions Education: A Critical Narrative Synthesis

Sukhera Javeed Western University

Wodzinski Michael Western University

Rehman Maham Western University

Electronic publication date: 2019 Apr 3

JOURNAL INFORMATION
==============================
NLM Title Abbreviation: Can Med Educ J
Journal ID: Can Med Educ J
NLM Journal ID: 101560935
Journal ID: CMEJ

Canadian Medical Education Journal

EISSN: 1923-1202
Publisher: University of Saskatchewan

ARTICLE INFORMATION
==============================
Subjects: Oa - 8 – 5

Mistreatment, abuse, unprofessionalism and erosive behaviours: How do medical learners make sense of negative interpersonal behaviour in the clinical workplace?

Vanstone Meredith McMaster University

Alice Cavanagh McMaster University

Emily Block McMaster University

Amanda Bell McMaster University

Catherine Connelly McMaster University

Margo Mountjoy McMaster University

Grierson Lawrence McMaster University

Electronic publication date: 2019 Apr 3

JOURNAL INFORMATION
==============================
NLM Title Abbreviation: Can Med Educ J
Journal ID: Can Med Educ J
NLM Journal ID: 101560935
Journal ID: CMEJ

Canadian Medical Education Journal

EISSN: 1923-1202
Publisher: University of Saskatchewan

ARTICLE INFORMATION
==============================
Subjects: Oa - 8 – 6

A Human Library Intervention to Address Bias towards LGBTQ Individuals

Anderson Michelle University of Alberta

Fehr Derek University of Alberta

Goez Helly University of Alberta

Rodger Joanne University of Alberta

Daniels Lia University of Alberta

Lai Hollis University of Alberta

Daniels Vijay University of Alberta

Hillier Tracey University of Alberta

Electronic publication date: 2019 Apr 3

JOURNAL INFORMATION
==============================
NLM Title Abbreviation: Can Med Educ J
Journal ID: Can Med Educ J
NLM Journal ID: 101560935
Journal ID: CMEJ

Canadian Medical Education Journal

EISSN: 1923-1202
Publisher: University of Saskatchewan

ARTICLE INFORMATION
==============================
Subjects: Ob - 1 – 1

Improving residents' confidence and accuracy in making clinical judgement: Evidence from a multi-institutional simulation based study

Thoma Bren University of Saskatchewan

Dalgarno Nancy Queen’s University

Egan Rylan Queen’s University

Gu Jeffrey University of Saskatchewan

McColl Tamara University of Manitoba

Chaplin Tim Queen’s University

Cofie Nicholas Queen’s University

Electronic publication date: 2019 Apr 3

JOURNAL INFORMATION
==============================
NLM Title Abbreviation: Can Med Educ J
Journal ID: Can Med Educ J
NLM Journal ID: 101560935
Journal ID: CMEJ

Canadian Medical Education Journal

EISSN: 1923-1202
Publisher: University of Saskatchewan

ARTICLE INFORMATION
==============================
Subjects: Ob - 1 – 2

Multiple Choice exams at the UGME level: Ensuring quality pre- and post-exam administration

St-Onge Christina Université de Sherbrooke

Young Meredith McGill

Renaud Jean-Sébastien Université Laval

Drescher Olivia Université Laval

Cummings Beth-Ann McGill

Varpio Lara Uniformed Services University of the Health Sciences

Electronic publication date: 2019 Apr 3

JOURNAL INFORMATION
==============================
NLM Title Abbreviation: Can Med Educ J
Journal ID: Can Med Educ J
NLM Journal ID: 101560935
Journal ID: CMEJ

Canadian Medical Education Journal

EISSN: 1923-1202
Publisher: University of Saskatchewan

ARTICLE INFORMATION
==============================
Subjects: Ob - 1 – 3

Dual-Purposing Assessment Data: The Impact of Assessment Purpose on Rater Behaviour in the Assessment of Competence

Tavares Walter University of Toronto

Young Meredith McGill

Gauthier Genevieve Université de Sherbrooke

St-Onge Christina Université de Sherbrooke

Electronic publication date: 2019 Apr 3

JOURNAL INFORMATION
==============================
NLM Title Abbreviation: Can Med Educ J
Journal ID: Can Med Educ J
NLM Journal ID: 101560935
Journal ID: CMEJ

Canadian Medical Education Journal

EISSN: 1923-1202
Publisher: University of Saskatchewan

ARTICLE INFORMATION
==============================
Subjects: Ob - 1 – 4

An evaluative analysis of rater variability in Modified Personal Interviews for the MD Program, University of Toronto

Tu Yuxin University of Toronto

Latter David University of Toronto

(Mahan) Kulasegaram Kulamakan University of Toronto

Lee Hana University of Toronto

Electronic publication date: 2019 Apr 3

JOURNAL INFORMATION
==============================
NLM Title Abbreviation: Can Med Educ J
Journal ID: Can Med Educ J
NLM Journal ID: 101560935
Journal ID: CMEJ

Canadian Medical Education Journal

EISSN: 1923-1202
Publisher: University of Saskatchewan

ARTICLE INFORMATION
==============================
Subjects: Ob - 1 – 5

Taken out of context: hazards in the interpretation of written assessment comments

Ginsburg Shiphra University of Toronto

Watling Christopher Western University

Gingerich Andrea University of British Columbia

Kogan Jennifer University of Pennsylvania

Watling Chris Western University, Meghan Lynch University of Toronto

Electronic publication date: 2019 Apr 3

JOURNAL INFORMATION
==============================
NLM Title Abbreviation: Can Med Educ J
Journal ID: Can Med Educ J
NLM Journal ID: 101560935
Journal ID: CMEJ

Canadian Medical Education Journal

EISSN: 1923-1202
Publisher: University of Saskatchewan

ARTICLE INFORMATION
==============================
Subjects: Ob - 1 – 6

Scenes, symbols and social roles: OSCE performativity and identities

Gormley Gerry QUB

Corrigan Mairead QUB

Johnston Jennifer QUB

Electronic publication date: 2019 Apr 3

JOURNAL INFORMATION
==============================
NLM Title Abbreviation: Can Med Educ J
Journal ID: Can Med Educ J
NLM Journal ID: 101560935
Journal ID: CMEJ

Canadian Medical Education Journal

EISSN: 1923-1202
Publisher: University of Saskatchewan

ARTICLE INFORMATION
==============================
Subjects: Ob - 2 – 1

Towards improved entrustability judgments through exploration of supervisors' real-time assessment

Luu Kimberly University of British Columbia

Eva Kevin University of British Columbia

Chadha Neil University of British Columbia

Sidhu Ravi University of British Columbia

Electronic publication date: 2019 Apr 3

JOURNAL INFORMATION
==============================
NLM Title Abbreviation: Can Med Educ J
Journal ID: Can Med Educ J
NLM Journal ID: 101560935
Journal ID: CMEJ

Canadian Medical Education Journal

EISSN: 1923-1202
Publisher: University of Saskatchewan

ARTICLE INFORMATION
==============================
Subjects: Ob - 2 – 2

Entrustment, competence and tensions in assessment: the realities of entrustment in Internal Medicine

Rassos James University of Toronto

Melvin Lindsay University of Toronto

Stroud Lynfa University of Toronto

Ginsburg Shiphra University of Toronto

Electronic publication date: 2019 Apr 3

JOURNAL INFORMATION
==============================
NLM Title Abbreviation: Can Med Educ J
Journal ID: Can Med Educ J
NLM Journal ID: 101560935
Journal ID: CMEJ

Canadian Medical Education Journal

EISSN: 1923-1202
Publisher: University of Saskatchewan

ARTICLE INFORMATION
==============================
Subjects: Ob - 2 – 3

Some Assembly Required: ‘Problematic evidence’ and the interpretative work of Clinical Competency Committees

Pack Rachael Western University

Cristancho Sayra Western University

Watling Chris Western University

Lingard Lorelei Western University

Chahine Saad Western University

Electronic publication date: 2019 Apr 3

JOURNAL INFORMATION
==============================
NLM Title Abbreviation: Can Med Educ J
Journal ID: Can Med Educ J
NLM Journal ID: 101560935
Journal ID: CMEJ

Canadian Medical Education Journal

EISSN: 1923-1202
Publisher: University of Saskatchewan

ARTICLE INFORMATION
==============================
Subjects: Ob - 2 – 4

Competency-based medical education: setting an evidence-informed research agenda

Brydges Ryan University of Toronto

Boyd Victoria University of Toronto

Ginsburg Shiphra University of Toronto

Stroud Lynfa University of Toronto

Kuper Ayelet University of Toronto

Tavares Walter University of Toronto

Electronic publication date: 2019 Apr 3

JOURNAL INFORMATION
==============================
NLM Title Abbreviation: Can Med Educ J
Journal ID: Can Med Educ J
NLM Journal ID: 101560935
Journal ID: CMEJ

Canadian Medical Education Journal

EISSN: 1923-1202
Publisher: University of Saskatchewan

ARTICLE INFORMATION
==============================
Subjects: Ob - 2 – 5

Developing academic advisors and competency committee members competencies: A grassroots community approach to Faculty Development

van Wylick Richard Queen’s University

Stockley Denise Queen’s University

Dagnone Damon Queen’s University

Garton Kendall Queen’s University

Soleas Eleftherios Queen’s University

Harle Ingrid Queen’s University

Electronic publication date: 2019 Apr 3

JOURNAL INFORMATION
==============================
NLM Title Abbreviation: Can Med Educ J
Journal ID: Can Med Educ J
NLM Journal ID: 101560935
Journal ID: CMEJ

Canadian Medical Education Journal

EISSN: 1923-1202
Publisher: University of Saskatchewan

ARTICLE INFORMATION
==============================
Subjects: Ob - 2 – 6

Anticipating Competence by Design: faculty and resident perspectives

Lord Jason University of Calgary

Gaudet Jonathan University of Calgary

Eng Reuben University of Calgary, Rachel Ellaway University of Calgary

Adegbesan Cinde University of Calgary

Pokharel Surakshya University of Calgary

Millar Kelly University of Calgary

Electronic publication date: 2019 Apr 3

JOURNAL INFORMATION
==============================
NLM Title Abbreviation: Can Med Educ J
Journal ID: Can Med Educ J
NLM Journal ID: 101560935
Journal ID: CMEJ

Canadian Medical Education Journal

EISSN: 1923-1202
Publisher: University of Saskatchewan

ARTICLE INFORMATION
==============================
Subjects: Ob - 3 – 1

Vocal Coaching Through Song: Exploring the Impact of Pedagogical Vocal Training on Clinical Interactions

Radmacher Bruce University of Saskatchewan

Malin Greg University of Saskatchewan

McCulloch Andrea Voice Instructor

Electronic publication date: 2019 Apr 3

JOURNAL INFORMATION
==============================
NLM Title Abbreviation: Can Med Educ J
Journal ID: Can Med Educ J
NLM Journal ID: 101560935
Journal ID: CMEJ

Canadian Medical Education Journal

EISSN: 1923-1202
Publisher: University of Saskatchewan

ARTICLE INFORMATION
==============================
Subjects: Ob - 3 – 2

Writing as thinking: A study of documentation practices and their implications for medical education

Bowker Dillon McMaster University

Goldszmidt Mark Western University

Torti Jacqueline Western University

Electronic publication date: 2019 Apr 3

JOURNAL INFORMATION
==============================
NLM Title Abbreviation: Can Med Educ J
Journal ID: Can Med Educ J
NLM Journal ID: 101560935
Journal ID: CMEJ

Canadian Medical Education Journal

EISSN: 1923-1202
Publisher: University of Saskatchewan

ARTICLE INFORMATION
==============================
Subjects: Ob - 3 – 3

Embodied empathy, a phenomenological study of physician touch.

Kelly Martina University of Calgary

Svrcek Clark University of Calgary

Dornan Tim Queen's University Belfast

Electronic publication date: 2019 Apr 3

JOURNAL INFORMATION
==============================
NLM Title Abbreviation: Can Med Educ J
Journal ID: Can Med Educ J
NLM Journal ID: 101560935
Journal ID: CMEJ

Canadian Medical Education Journal

EISSN: 1923-1202
Publisher: University of Saskatchewan

ARTICLE INFORMATION
==============================
Subjects: Ob - 3 – 4

Learning to take a sexual history in the Pediatric population: A Canadian medical school clinical skills curriculum

Hudson Alexandra University of Alberta

Blake Kim Dalhousie University

Electronic publication date: 2019 Apr 3

JOURNAL INFORMATION
==============================
NLM Title Abbreviation: Can Med Educ J
Journal ID: Can Med Educ J
NLM Journal ID: 101560935
Journal ID: CMEJ

Canadian Medical Education Journal

EISSN: 1923-1202
Publisher: University of Saskatchewan

ARTICLE INFORMATION
==============================
Subjects: Ob - 3 – 5

Best Practices in Medicine (BPiM): Right-Sizing Laboratory and Diagnostic Imaging Test Orders at a Community Teaching Hospital

Jackman Mallory University of Toronto

Wooster Elizabeth OISE/University of Toronto

Tron Victor University of Toronto

Kapur Ajay University of Toronto

Seth Rishie University of Toronto

Maniate Jerry University of Ottawa

Pasic Maria University of Toronto

Kherani Raheem University of British Columbia

Mulsant Sharon University of Toronto

Arab-O'Brien Donna University of Toronto

Barnes Craig St Joseph's Health Centre

Taher Jennifer University of Toronto

Electronic publication date: 2019 Apr 3

JOURNAL INFORMATION
==============================
NLM Title Abbreviation: Can Med Educ J
Journal ID: Can Med Educ J
NLM Journal ID: 101560935
Journal ID: CMEJ

Canadian Medical Education Journal

EISSN: 1923-1202
Publisher: University of Saskatchewan

ARTICLE INFORMATION
==============================
Subjects: Ob - 3 – 6

Developing and Implementing the Best Practices in Medicine (BPiM) Framework: a "Right-Sizing" Test Utilization Campaign

Wooster Elizabeth OISE/University of Toronto

Jackman Mallory University of Toronto, Jerry Maniate University of Ottawa

Kapur Ajay University of Toronto

Barnes Craig St Joseph's Health Centre

Arab-O'Brien Donna University of Toronto

Pasic Maria University of Toronto

Tron Victor University of Toronto

Seth Rishie University of Toronto

Kherani Raheem University of British Columbia

Mulsant Sharon University of Toronto

Taher Jennifer University of Toronto

Electronic publication date: 2019 Apr 3

JOURNAL INFORMATION
==============================
NLM Title Abbreviation: Can Med Educ J
Journal ID: Can Med Educ J
NLM Journal ID: 101560935
Journal ID: CMEJ

Canadian Medical Education Journal

EISSN: 1923-1202
Publisher: University of Saskatchewan

ARTICLE INFORMATION
==============================
Subjects: Ob - 4 – 1

Addressing the Opioid Crisis: Evaluating the Safer Opioid Prescribing Program (SOP)

Hsu Justin University of Toronto

Sud Abhimanyu University of Toronto

Doukas Kathleen University of Toronto

Miatello Amber University of Toronto

Paton Morag University of Toronto

Electronic publication date: 2019 Apr 3

JOURNAL INFORMATION
==============================
NLM Title Abbreviation: Can Med Educ J
Journal ID: Can Med Educ J
NLM Journal ID: 101560935
Journal ID: CMEJ

Canadian Medical Education Journal

EISSN: 1923-1202
Publisher: University of Saskatchewan

ARTICLE INFORMATION
==============================
Subjects: Ob - 4 – 2

CPD by the Minute: an innovative use of mobile technology to improve continuing medical education and physician self-assessment

Wiljer David University of Toronto

Slinger Peter University of Toronto

Rotstein Alexandra University of Toronto

Charow Rebecca University Health Network

MacLellan Brent University of Toronto, Connor Moore University Health Network

Papadakos Tina University Health Network

Giuliani Meredith University of Toronto

Electronic publication date: 2019 Apr 3

JOURNAL INFORMATION
==============================
NLM Title Abbreviation: Can Med Educ J
Journal ID: Can Med Educ J
NLM Journal ID: 101560935
Journal ID: CMEJ

Canadian Medical Education Journal

EISSN: 1923-1202
Publisher: University of Saskatchewan

ARTICLE INFORMATION
==============================
Subjects: Ob - 4 – 3

Rethinking CPD: Building a simulation program through collaboration

Wade Patricia Fédération des médecins spécialistes du Québec

J. Daniel Sam Fédération des médecins spécialistes du Québec

Bouchard Marie-Josée Fédération des médecins spécialistes du Québec

Tremblay Martin Fédération des médecins spécialistes du Québec

Electronic publication date: 2019 Apr 3

JOURNAL INFORMATION
==============================
NLM Title Abbreviation: Can Med Educ J
Journal ID: Can Med Educ J
NLM Journal ID: 101560935
Journal ID: CMEJ

Canadian Medical Education Journal

EISSN: 1923-1202
Publisher: University of Saskatchewan

ARTICLE INFORMATION
==============================
Subjects: Ob - 4 – 4

A Needs Assessment to Support Implementation of the Electronic Medical Record (EMR) in Newfoundland and Labrador (NL)

Snow Pamela Memorial – University of Newfoundland

Curran Vernon Memorial – University of Newfoundland

Fleet Lisa Memorial – University of Newfoundland

Electronic publication date: 2019 Apr 3

JOURNAL INFORMATION
==============================
NLM Title Abbreviation: Can Med Educ J
Journal ID: Can Med Educ J
NLM Journal ID: 101560935
Journal ID: CMEJ

Canadian Medical Education Journal

EISSN: 1923-1202
Publisher: University of Saskatchewan

ARTICLE INFORMATION
==============================
Subjects: Ob - 4 – 5

Physician Continuous Quality Improvement in Rural British Columbia: A Qualitative Analysis

Bluman Bob University of British Columbia

Lynn Brenna University of British Columbia

Leung Dilys University of British Columbia

Born Dawson University of British Columbia

Markham Ray University of British Columbia

Electronic publication date: 2019 Apr 3

JOURNAL INFORMATION
==============================
NLM Title Abbreviation: Can Med Educ J
Journal ID: Can Med Educ J
NLM Journal ID: 101560935
Journal ID: CMEJ

Canadian Medical Education Journal

EISSN: 1923-1202
Publisher: University of Saskatchewan

ARTICLE INFORMATION
==============================
Subjects: Ob - 4 – 6

Reaching Far and Wide: Assessing the Uptake of British Columbia (BC) Centre for Excellence in HIV/AIDS (BC-CfE) Multimodal Learning Opportunities

Guillemi Silvia British Columbia Centre for Excellence in HIV/AIDS

Puskas Cathy British Columbia Centre for Excellence in HIV/AIDS

Koleszar Karah British Columbia Centre for Excellence in HIV/AIDS

Electronic publication date: 2019 Apr 3

JOURNAL INFORMATION
==============================
NLM Title Abbreviation: Can Med Educ J
Journal ID: Can Med Educ J
NLM Journal ID: 101560935
Journal ID: CMEJ

Canadian Medical Education Journal

EISSN: 1923-1202
Publisher: University of Saskatchewan

ARTICLE INFORMATION
==============================
Subjects: Ob - 5 – 1

Aligning Requirements of Training and Assessment in Radiation Planning in the Era of Competency-Based Medical Education

de Metz Catherine Queen’s University

Kalyvas Maria Queen’s University

Moideen Nikitha Queen’s University

Soleas Eleftherios Queen’s University

Dalgarno Nancy Queen’s University

Egan Rylan Queen’s University

Coderre-Ball Angela Queen’s University

Electronic publication date: 2019 Apr 3

JOURNAL INFORMATION
==============================
NLM Title Abbreviation: Can Med Educ J
Journal ID: Can Med Educ J
NLM Journal ID: 101560935
Journal ID: CMEJ

Canadian Medical Education Journal

EISSN: 1923-1202
Publisher: University of Saskatchewan

ARTICLE INFORMATION
==============================
Subjects: Ob - 5 – 2

A systematic review of content rating methods for courses in medical schools

D'Eon Marcel University of Saskatchewan

Harris June Memorial – University of Newfoundland

Wright Claire Chaminade University

Bull Harold University of Saskatchewan

Malin Greg University of Saskatchewan

Anderson Kyle University of Saskatchewan

Sakai Damon University of Hawaii

Domes Trustin University of Saskatchewan

Premkumar Kalyani University of Saskatchewan

Electronic publication date: 2019 Apr 3

JOURNAL INFORMATION
==============================
NLM Title Abbreviation: Can Med Educ J
Journal ID: Can Med Educ J
NLM Journal ID: 101560935
Journal ID: CMEJ

Canadian Medical Education Journal

EISSN: 1923-1202
Publisher: University of Saskatchewan

ARTICLE INFORMATION
==============================
Subjects: Ob - 5 – 3

When Strangers MEET: Making Every Encounter Therapeutic

Tan Adrienne University of Toronto

Chaudhary Zarah University of Toronto

Mylopoulos Maria University of Toronto

Sockalingam Sanjeev University of Toronto

Electronic publication date: 2019 Apr 3

JOURNAL INFORMATION
==============================
NLM Title Abbreviation: Can Med Educ J
Journal ID: Can Med Educ J
NLM Journal ID: 101560935
Journal ID: CMEJ

Canadian Medical Education Journal

EISSN: 1923-1202
Publisher: University of Saskatchewan

ARTICLE INFORMATION
==============================
Subjects: Ob - 5 – 4

Competency-based Internal Medicine Point-of-Care Ultrasound Super User Program

Brotherston Drew University of Calgary

Desy Janeve University of Calgary

Mintz Marcy University of Calgary

Ma Irene University of Calgary

Electronic publication date: 2019 Apr 3

JOURNAL INFORMATION
==============================
NLM Title Abbreviation: Can Med Educ J
Journal ID: Can Med Educ J
NLM Journal ID: 101560935
Journal ID: CMEJ

Canadian Medical Education Journal

EISSN: 1923-1202
Publisher: University of Saskatchewan

ARTICLE INFORMATION
==============================
Subjects: Ob - 5 – 5

Responding to requests for hastened death: Promoting quality care and education in the era of medical assistance in dying

Christy Kayonne McMaster University

Vanstone Meredith McMaster University

Grierson Lawrence McMaster University

Shadd Joshua McMaster University

Farag Alexandra McMaster University

Patel Tejal McMaster University

Electronic publication date: 2019 Apr 3

JOURNAL INFORMATION
==============================
NLM Title Abbreviation: Can Med Educ J
Journal ID: Can Med Educ J
NLM Journal ID: 101560935
Journal ID: CMEJ

Canadian Medical Education Journal

EISSN: 1923-1202
Publisher: University of Saskatchewan

ARTICLE INFORMATION
==============================
Subjects: Ob - 5 – 6

A Theory-based Curriculum, a Medical Student, and a CBL Tutor Walk into a Bar: Evaluating Curriculum Enactment, Adaptation, and Resistance during Reform

Onyura Betty University of Toronto

Lazor Jana University of Toronto

Jugnundan Sechiv University of Toronto

Barned Claudia McGill

Boyd Victoria University of Toronto

Electronic publication date: 2019 Apr 3

JOURNAL INFORMATION
==============================
NLM Title Abbreviation: Can Med Educ J
Journal ID: Can Med Educ J
NLM Journal ID: 101560935
Journal ID: CMEJ

Canadian Medical Education Journal

EISSN: 1923-1202
Publisher: University of Saskatchewan

ARTICLE INFORMATION
==============================
Subjects: Ob - 6 – 1

The Impact of Self-Reported Socioeconomic Disadvantage and Education-Occupation Classification on MD Application Metrics at the University of Calgary

Walker Ian University of Calgary

Cayer Shannon University of Calgary

Electronic publication date: 2019 Apr 3

JOURNAL INFORMATION
==============================
NLM Title Abbreviation: Can Med Educ J
Journal ID: Can Med Educ J
NLM Journal ID: 101560935
Journal ID: CMEJ

Canadian Medical Education Journal

EISSN: 1923-1202
Publisher: University of Saskatchewan

ARTICLE INFORMATION
==============================
Subjects: Ob - 6 – 2

Experiences of Black physicians/physician trainees in medicine in Ontario

Mpalirwa Joseph University of Toronto

Lofters Aisha University of Toronto

Mpalirwa Joseph University of Toronto

Nnorom Onye University of Toronto

Hanson Mark University of Toronto

Electronic publication date: 2019 Apr 3

JOURNAL INFORMATION
==============================
NLM Title Abbreviation: Can Med Educ J
Journal ID: Can Med Educ J
NLM Journal ID: 101560935
Journal ID: CMEJ

Canadian Medical Education Journal

EISSN: 1923-1202
Publisher: University of Saskatchewan

ARTICLE INFORMATION
==============================
Subjects: Ob - 6 – 3

Islamophobia in Residency

Balakrishna Anita University of Toronto

Babcock Glenys University of Toronto

Ruetalo Mariela University of Toronto

Bandiera Glen University of Toronto

Robinson Lisa University of Toronto

Electronic publication date: 2019 Apr 3

JOURNAL INFORMATION
==============================
NLM Title Abbreviation: Can Med Educ J
Journal ID: Can Med Educ J
NLM Journal ID: 101560935
Journal ID: CMEJ

Canadian Medical Education Journal

EISSN: 1923-1202
Publisher: University of Saskatchewan

ARTICLE INFORMATION
==============================
Subjects: Ob - 6 – 4

Equity: Colliding Perceptions of Advantage among Medical Students

Babcock Glenys University of Toronto

Ruetalo Mariela University of Toronto

Nnorom Onye University of Toronto

Balakrishna Anita University of Toronto

Robinson Lisa University of Toronto

Electronic publication date: 2019 Apr 3

JOURNAL INFORMATION
==============================
NLM Title Abbreviation: Can Med Educ J
Journal ID: Can Med Educ J
NLM Journal ID: 101560935
Journal ID: CMEJ

Canadian Medical Education Journal

EISSN: 1923-1202
Publisher: University of Saskatchewan

ARTICLE INFORMATION
==============================
Subjects: Ob - 6 – 5

"Visibility as survival, visibility versus survival": The experiences of trans and gender non-conforming medical students in balancing professionalism, authenticity, and safety

Butler Kat McMaster University

Veltman Albina McMaster University

Yak Adryen University of Toronto

Electronic publication date: 2019 Apr 3

JOURNAL INFORMATION
==============================
NLM Title Abbreviation: Can Med Educ J
Journal ID: Can Med Educ J
NLM Journal ID: 101560935
Journal ID: CMEJ

Canadian Medical Education Journal

EISSN: 1923-1202
Publisher: University of Saskatchewan

ARTICLE INFORMATION
==============================
Subjects: Ob - 6 – 6

What's in a name: An analysis of ethnic origins of patient and provider names in Case-Based Learning

Berditchevskaia Inna University of Toronto

Balbaa* Amira University of Toronto

Bowden* Sylvie University of Toronto

Freeman* Sarah University of Toronto

Kirubarajan* Abirami University of Toronto

Klostermann* Natalie University of Toronto

Naguib* Mariam University of Toronto

Lazor Jana University of Toronto

Naguib Mariam University of Toronto

Electronic publication date: 2019 Apr 3

JOURNAL INFORMATION
==============================
NLM Title Abbreviation: Can Med Educ J
Journal ID: Can Med Educ J
NLM Journal ID: 101560935
Journal ID: CMEJ

Canadian Medical Education Journal

EISSN: 1923-1202
Publisher: University of Saskatchewan

ARTICLE INFORMATION
==============================
Subjects: Ob - 7 – 1

Identifying hawkish and dovish faculty assessors in anesthesiology clinical assessments

Fleming Melinda Queen’s University

Dalgarno Nancy Queen’s University

Cofie Nicholas Queen’s University

McMullen Michael Queen’s University

Egan Rylan Queen’s University

Electronic publication date: 2019 Apr 3

JOURNAL INFORMATION
==============================
NLM Title Abbreviation: Can Med Educ J
Journal ID: Can Med Educ J
NLM Journal ID: 101560935
Journal ID: CMEJ

Canadian Medical Education Journal

EISSN: 1923-1202
Publisher: University of Saskatchewan

ARTICLE INFORMATION
==============================
Subjects: Ob - 7 – 2

Supporting Canadian Athletic Therapy Supervisors in their Clinical Educator Role

Owen Jeffrey University of Calgary

Palacios Maria Mackay University of Calgary

Oddone Paolucci Elizabeth University of Calgary

Lafave Mark Mount Royal University

Yeo Michelle Mount Royal University

Electronic publication date: 2019 Apr 3

JOURNAL INFORMATION
==============================
NLM Title Abbreviation: Can Med Educ J
Journal ID: Can Med Educ J
NLM Journal ID: 101560935
Journal ID: CMEJ

Canadian Medical Education Journal

EISSN: 1923-1202
Publisher: University of Saskatchewan

ARTICLE INFORMATION
==============================
Subjects: Ob - 7 – 3

Essentials of Teaching: Interprofessional Faculty Development in the Clinical and Classroom Settings

van Wylick Richard Queen’s University

De Sousa Mikaila Queen’s University

Harle Ingrid Queen’s University

Soleas Eleftherios Queen’s University

Carpenter Jennifer Queen’s University

McDiarmid Laura Queen’s University

Electronic publication date: 2019 Apr 3

JOURNAL INFORMATION
==============================
NLM Title Abbreviation: Can Med Educ J
Journal ID: Can Med Educ J
NLM Journal ID: 101560935
Journal ID: CMEJ

Canadian Medical Education Journal

EISSN: 1923-1202
Publisher: University of Saskatchewan

ARTICLE INFORMATION
==============================
Subjects: Ob - 7 – 4

Faculty development to support curriculum renewal: An aligned approach

Lazor Jana University of Toronto

Innes Lori University of Toronto

Bell Jennifer University of Toronto

Electronic publication date: 2019 Apr 3

JOURNAL INFORMATION
==============================
NLM Title Abbreviation: Can Med Educ J
Journal ID: Can Med Educ J
NLM Journal ID: 101560935
Journal ID: CMEJ

Canadian Medical Education Journal

EISSN: 1923-1202
Publisher: University of Saskatchewan

ARTICLE INFORMATION
==============================
Subjects: Ob - 7 – 5

Academic Productivity: Valuing the Clinician Teacher

Freeman Risa University of Toronto

White David University of Toronto

Paton Morag University of Toronto

Krueger Paul University of Toronto

Tannenbaum David University of Toronto

Heisey Ruth University of Toronto

Murdoch Stuart University of Toronto

Kulasegaram Kulamakan University of Toronto

Electronic publication date: 2019 Apr 3

JOURNAL INFORMATION
==============================
NLM Title Abbreviation: Can Med Educ J
Journal ID: Can Med Educ J
NLM Journal ID: 101560935
Journal ID: CMEJ

Canadian Medical Education Journal

EISSN: 1923-1202
Publisher: University of Saskatchewan

ARTICLE INFORMATION
==============================
Subjects: Ob - 7 – 6

The Art of the Possible: What have we learned about creating an education scholarship grant program?

Freeman Risa University of Toronto

Penciner Rick University of Toronto

Bordman Risa University of Toronto

Ellis Rachel University of Toronto

Forte Milena University of Toronto

Ghavam-Rassoul Abbas University of Toronto

Kulasegaram Kulamakan University of Toronto

Nutik Melissa University of Toronto

Nyhof-Young Joyce University of Toronto

Onyura Betty University of Toronto

Whitehead Cynthia University of Toronto

Wright Sarah University of Toronto

Woods Nicole University of Toronto

Antao Viola University of Toronto

Electronic publication date: 2019 Apr 3

JOURNAL INFORMATION
==============================
NLM Title Abbreviation: Can Med Educ J
Journal ID: Can Med Educ J
NLM Journal ID: 101560935
Journal ID: CMEJ

Canadian Medical Education Journal

EISSN: 1923-1202
Publisher: University of Saskatchewan

ARTICLE INFORMATION
==============================
Subjects: Ob - 8 – 1

Towards Sexual Health for Every Body? Exploring First Year Medical Students' Experiences with an LGBTQ2S-inclusive Sexual History Education Session

Song Kaiwen University of Toronto

Biro Laurence University of Toronto

Nyhof-Young Joyce University of Toronto

Wong David University of Toronto

Electronic publication date: 2019 Apr 3

JOURNAL INFORMATION
==============================
NLM Title Abbreviation: Can Med Educ J
Journal ID: Can Med Educ J
NLM Journal ID: 101560935
Journal ID: CMEJ

Canadian Medical Education Journal

EISSN: 1923-1202
Publisher: University of Saskatchewan

ARTICLE INFORMATION
==============================
Subjects: Ob - 8 – 2

Building the "good" doctor: Changing patterns of humanism in medical students

Holland Alyson McMaster University

Platt Elyse McMaster University

Electronic publication date: 2019 Apr 3

JOURNAL INFORMATION
==============================
NLM Title Abbreviation: Can Med Educ J
Journal ID: Can Med Educ J
NLM Journal ID: 101560935
Journal ID: CMEJ

Canadian Medical Education Journal

EISSN: 1923-1202
Publisher: University of Saskatchewan

ARTICLE INFORMATION
==============================
Subjects: Ob - 8 – 3

Wilderness Education - Becoming an Expert Teacher at the Distributed Medical Education Campus

Didyk Nicole McMaster University

Vanstone Meredith McMaster University

Electronic publication date: 2019 Apr 3

JOURNAL INFORMATION
==============================
NLM Title Abbreviation: Can Med Educ J
Journal ID: Can Med Educ J
NLM Journal ID: 101560935
Journal ID: CMEJ

Canadian Medical Education Journal

EISSN: 1923-1202
Publisher: University of Saskatchewan

ARTICLE INFORMATION
==============================
Subjects: Ob - 8 – 4

Consulting the Oracle: Heterogeneity and Health of Distributed Medical Education in Canada - Results of a National Delphi Study

Murray PhD John University of Manitoba

Alexiadis-Brown MA Peggy Dalhousie University

Hatcher MD MSc FCMF Sharon Université de Sherbrooke

Larouche PhD Catherine Universite du Quebec a Chicoutimi

Penner MD FRCPC Charles University of Manitoba

Electronic publication date: 2019 Apr 3

JOURNAL INFORMATION
==============================
NLM Title Abbreviation: Can Med Educ J
Journal ID: Can Med Educ J
NLM Journal ID: 101560935
Journal ID: CMEJ

Canadian Medical Education Journal

EISSN: 1923-1202
Publisher: University of Saskatchewan

ARTICLE INFORMATION
==============================
Subjects: Ob - 8 – 5

Perspectives of Urban First Year Medical Students in Choosing Practice Location

Malhi Rebecca University of Calgary

Konkin Jill University of Alberta

Myhre Douglas University of Calgary

Smith-Windsor Tom University of Saskatchewan

Woloschuk Wayne University of Calgary

Lemoine Daniel University of Alberta

Electronic publication date: 2019 Apr 3

JOURNAL INFORMATION
==============================
NLM Title Abbreviation: Can Med Educ J
Journal ID: Can Med Educ J
NLM Journal ID: 101560935
Journal ID: CMEJ

Canadian Medical Education Journal

EISSN: 1923-1202
Publisher: University of Saskatchewan

ARTICLE INFORMATION
==============================
Subjects: Ob - 8 – 6

Probability of an offer based on pre- and post-interview rank at McMaster University's Undergraduate MD Program

C. Burgess Raquel McMaster University

Mountjoy Margo McMaster University

Vanstone Meredith McMaster University

E. M. Grierson Lawrence McMaster University

Electronic publication date: 2019 Apr 3

JOURNAL INFORMATION
==============================
NLM Title Abbreviation: Can Med Educ J
Journal ID: Can Med Educ J
NLM Journal ID: 101560935
Journal ID: CMEJ

Canadian Medical Education Journal

EISSN: 1923-1202
Publisher: University of Saskatchewan

ARTICLE INFORMATION
==============================
Subjects: Oc - 1 – 1

Longitudinal examination of residents' development of surgical competence using a national formative test

Gomez-Garibello Carlos McGill

Wagner Maryam McGill

Fata Paola McGill

Vair Brock Dalhousie University

Electronic publication date: 2019 Apr 3

JOURNAL INFORMATION
==============================
NLM Title Abbreviation: Can Med Educ J
Journal ID: Can Med Educ J
NLM Journal ID: 101560935
Journal ID: CMEJ

Canadian Medical Education Journal

EISSN: 1923-1202
Publisher: University of Saskatchewan

ARTICLE INFORMATION
==============================
Subjects: Oc - 1 – 2

Moving from the operating room to the exam room: The behavioural and cognitive skills used by clinicians to develop a formative exam for residents in General Surgery

Wagner Maryam McGill

Gomez Garibello Carlos McGill

Fata Paola McGill

Vair Brock Dalhousie University

Electronic publication date: 2019 Apr 3

JOURNAL INFORMATION
==============================
NLM Title Abbreviation: Can Med Educ J
Journal ID: Can Med Educ J
NLM Journal ID: 101560935
Journal ID: CMEJ

Canadian Medical Education Journal

EISSN: 1923-1202
Publisher: University of Saskatchewan

ARTICLE INFORMATION
==============================
Subjects: Oc - 1 – 3

Daily encounter cards are superior for student assessment compared to single rater in-training evaluation reports.

Johnson James University of Manitoba

Pinsk Maury University of Manitoba

Electronic publication date: 2019 Apr 3

JOURNAL INFORMATION
==============================
NLM Title Abbreviation: Can Med Educ J
Journal ID: Can Med Educ J
NLM Journal ID: 101560935
Journal ID: CMEJ

Canadian Medical Education Journal

EISSN: 1923-1202
Publisher: University of Saskatchewan

ARTICLE INFORMATION
==============================
Subjects: Oc - 1 – 4

Progress Test Predictive Analytics: Understanding Student Engagement with Assessment for Learning

Pan Pauline University of Toronto

Pittini Richard University of Toronto

Kulasegaram Kulamakan University of Toronto

Charles Tamica University of Toronto

Tu Yuxin University of Toronto

Tait Glendon University of Toronto

Howard Frazer University of Toronto

Rojas David University of Toronto

Electronic publication date: 2019 Apr 3

JOURNAL INFORMATION
==============================
NLM Title Abbreviation: Can Med Educ J
Journal ID: Can Med Educ J
NLM Journal ID: 101560935
Journal ID: CMEJ

Canadian Medical Education Journal

EISSN: 1923-1202
Publisher: University of Saskatchewan

ARTICLE INFORMATION
==============================
Subjects: Oc - 1 – 5

Introducing Progress Testing in the University of Toronto's New Foundations Curriculum: an Examination of Facts of Validity for the First Two Years of Assessments

Tu Yuxin University of Toronto

Pittini Richard University of Toronto

Tait Glendon University of Toronto

(Mahan) Kulasegaram Kulamakan University of Toronto

Tzanetos Katina University of Toronto

Pan Pauline University of Toronto

Electronic publication date: 2019 Apr 3

JOURNAL INFORMATION
==============================
NLM Title Abbreviation: Can Med Educ J
Journal ID: Can Med Educ J
NLM Journal ID: 101560935
Journal ID: CMEJ

Canadian Medical Education Journal

EISSN: 1923-1202
Publisher: University of Saskatchewan

ARTICLE INFORMATION
==============================
Subjects: Oc - 1 – 6

High-stakes testing accommodation: What is happening in the field? The Medical Council of Canada’s (MCC) Research on Testing Accommodations (TA)

Bartman Ilona Medical Council of Canada

Touchie Claire Medical Council of Canada

Condon Kathryn Medical Council of Canada

Electronic publication date: 2019 Apr 3

JOURNAL INFORMATION
==============================
NLM Title Abbreviation: Can Med Educ J
Journal ID: Can Med Educ J
NLM Journal ID: 101560935
Journal ID: CMEJ

Canadian Medical Education Journal

EISSN: 1923-1202
Publisher: University of Saskatchewan

ARTICLE INFORMATION
==============================
Subjects: Oc - 2 – 1

Feeling overwhelmed: Does CBME create graduates who are dependent upon coaching?

Oandasan Ivy The College of Family Physicians of Canada

Nardi Lorelei The College of Family Physicians of Canada

Kljujic Dragan The College of Family Physicians of Canada

Electronic publication date: 2019 Apr 3

JOURNAL INFORMATION
==============================
NLM Title Abbreviation: Can Med Educ J
Journal ID: Can Med Educ J
NLM Journal ID: 101560935
Journal ID: CMEJ

Canadian Medical Education Journal

EISSN: 1923-1202
Publisher: University of Saskatchewan

ARTICLE INFORMATION
==============================
Subjects: Oc - 2 – 2

Resource Optimization in Proficiency-Based Suturing Skills Training

Lemke Madeline Queen’s University

Lia Hillary Queen’s University

Gabinet-Equihua Alexander Queen’s University

Sheahan Guy Queen’s University

Winthrop Andrea Queen’s University

Mann Stephen Queen’s University

Fichtinger Gabor Queen’s University

Zevin Boris Queen’s University

Electronic publication date: 2019 Apr 3

JOURNAL INFORMATION
==============================
NLM Title Abbreviation: Can Med Educ J
Journal ID: Can Med Educ J
NLM Journal ID: 101560935
Journal ID: CMEJ

Canadian Medical Education Journal

EISSN: 1923-1202
Publisher: University of Saskatchewan

ARTICLE INFORMATION
==============================
Subjects: Oc - 2 – 3

Competency-based education frameworks across Canadian health professions and implications for multisource feedback

Wilbur Kerry University of British Columbia

Tong Brandon University of British Columbia

St. John Megan University of British Columbia

Li Emily University of British Columbia

Electronic publication date: 2019 Apr 3

JOURNAL INFORMATION
==============================
NLM Title Abbreviation: Can Med Educ J
Journal ID: Can Med Educ J
NLM Journal ID: 101560935
Journal ID: CMEJ

Canadian Medical Education Journal

EISSN: 1923-1202
Publisher: University of Saskatchewan

ARTICLE INFORMATION
==============================
Subjects: Oc - 2 – 4

Engaging Ophthalmology departmental stakeholders in shaping their program of resident assessment

Braund Heather Queen’s University

Baxter Stephanie Queen’s University

Dalgarno Nancy Queen’s University

McEwen Laura Queen’s University

Egan Rylan Queen’s University

Reid Mary-Anne Michigan State University

Electronic publication date: 2019 Apr 3

JOURNAL INFORMATION
==============================
NLM Title Abbreviation: Can Med Educ J
Journal ID: Can Med Educ J
NLM Journal ID: 101560935
Journal ID: CMEJ

Canadian Medical Education Journal

EISSN: 1923-1202
Publisher: University of Saskatchewan

ARTICLE INFORMATION
==============================
Subjects: Oc - 2 – 5

Resident Perceptions of Assessment and Feedback in Competency Based Medical Education (CBME)

Branfield Day Leora University of Toronto

Miles Amy University of Toronto

Melvin Lindsay University of Toronto

Ginsburg Shiphra University of Toronto

Electronic publication date: 2019 Apr 3

JOURNAL INFORMATION
==============================
NLM Title Abbreviation: Can Med Educ J
Journal ID: Can Med Educ J
NLM Journal ID: 101560935
Journal ID: CMEJ

Canadian Medical Education Journal

EISSN: 1923-1202
Publisher: University of Saskatchewan

ARTICLE INFORMATION
==============================
Subjects: Oc - 2 – 6

Examining trends in low stakes assessment information collected in a competency-based residency program

Ross Shelley University of Alberta

Humphries Paul University of Alberta

Donoff Mike University of Alberta

Schipper Shirley University of Alberta

Hamza Deena University of Alberta

Electronic publication date: 2019 Apr 3

JOURNAL INFORMATION
==============================
NLM Title Abbreviation: Can Med Educ J
Journal ID: Can Med Educ J
NLM Journal ID: 101560935
Journal ID: CMEJ

Canadian Medical Education Journal

EISSN: 1923-1202
Publisher: University of Saskatchewan

ARTICLE INFORMATION
==============================
Subjects: Od - 3 – 1

Which feedback do radiologists give on CanMEDS roles? What if residents ask?

Lafleur Alexandre Université Laval

Vincent Marc Université Laval

Côté Luc Université Laval

Simard Caroline Université Laval

Witteman Holly Université Laval

Martin-Zément Isabelle Université Laval

Electronic publication date: 2019 Apr 3

JOURNAL INFORMATION
==============================
NLM Title Abbreviation: Can Med Educ J
Journal ID: Can Med Educ J
NLM Journal ID: 101560935
Journal ID: CMEJ

Canadian Medical Education Journal

EISSN: 1923-1202
Publisher: University of Saskatchewan

ARTICLE INFORMATION
==============================
Subjects: Od - 3 – 2

Learning Conversations: A Reflective Analysis of Theoretical Roots and Manifestations of Feedback and Debriefing

Tavares Walter University of Toronto

Eppich Walter Northwestern University, Feinberg School of Medicine, Departments of Pediatrics and Medical Education

Cheng Adam University of Calgary

Miller Stephen Dalhousie University

Teunissen Pim School of Health Professions Education (SHE), Faculty of Health Medicine and Life Sciences, Maastricht University

Watling Chris Western University

Sargeant Joan Dalhousie University

Electronic publication date: 2019 Apr 3

JOURNAL INFORMATION
==============================
NLM Title Abbreviation: Can Med Educ J
Journal ID: Can Med Educ J
NLM Journal ID: 101560935
Journal ID: CMEJ

Canadian Medical Education Journal

EISSN: 1923-1202
Publisher: University of Saskatchewan

ARTICLE INFORMATION
==============================
Subjects: Od - 3 – 3

Perceptions of the Non-Physician Health Care Professional Providing Multisource Feedback in Competency Based Medical Education.

Miles Amy University of Toronto

Ginsburg Shiphra University of Toronto

Sibbald Matthew McMaster University

Tavares Walter University of Toronto

Khan Rabia University of Toronto

Watling Christopher Western University

Stroud Lynfa University of Toronto

Electronic publication date: 2019 Apr 3

JOURNAL INFORMATION
==============================
NLM Title Abbreviation: Can Med Educ J
Journal ID: Can Med Educ J
NLM Journal ID: 101560935
Journal ID: CMEJ

Canadian Medical Education Journal

EISSN: 1923-1202
Publisher: University of Saskatchewan

ARTICLE INFORMATION
==============================
Subjects: Od - 3 – 4

Bedside Observation and Feedback Practices in Internal Medicine: A Longitudinal Survey Study

Wang Michael McMaster University

Onizuka Kristyne McMaster University

Foster Christopher Western University

Khalil Ramy Queen’s University

Brandt Vegas Daniel McMaster University

Electronic publication date: 2019 Apr 3

JOURNAL INFORMATION
==============================
NLM Title Abbreviation: Can Med Educ J
Journal ID: Can Med Educ J
NLM Journal ID: 101560935
Journal ID: CMEJ

Canadian Medical Education Journal

EISSN: 1923-1202
Publisher: University of Saskatchewan

ARTICLE INFORMATION
==============================
Subjects: Od - 3 – 5

Physician Scorecards as a Novel Approach to Performance Feedback and Education: Should Quality be Negotiable?

Tang Brandon University of British Columbia

Lakhani Anand University of Toronto

Ginzburg Amir University of Toronto

Morra Dante University of Toronto

Electronic publication date: 2019 Apr 3

JOURNAL INFORMATION
==============================
NLM Title Abbreviation: Can Med Educ J
Journal ID: Can Med Educ J
NLM Journal ID: 101560935
Journal ID: CMEJ

Canadian Medical Education Journal

EISSN: 1923-1202
Publisher: University of Saskatchewan

ARTICLE INFORMATION
==============================
Subjects: Od - 3 – 6

Adapting comment prompts to improve narrative feedback for learners

Lai Hollis University of Alberta

Daniels Vijay University of Alberta

Hillier Tracey University of Alberta

Forbes Karen University of Alberta

Electronic publication date: 2019 Apr 3

JOURNAL INFORMATION
==============================
NLM Title Abbreviation: Can Med Educ J
Journal ID: Can Med Educ J
NLM Journal ID: 101560935
Journal ID: CMEJ

Canadian Medical Education Journal

EISSN: 1923-1202
Publisher: University of Saskatchewan

ARTICLE INFORMATION
==============================
Subjects: Oc - 4 – 1

Using photo-elicitation to capture patients' and physicians' perspectives about the Health Advocate role

Burm Sarah Western University

Watling Chris Western University

Cristancho Sayra Western University

LaDonna Kori University of Ottawa

Electronic publication date: 2019 Apr 3

JOURNAL INFORMATION
==============================
NLM Title Abbreviation: Can Med Educ J
Journal ID: Can Med Educ J
NLM Journal ID: 101560935
Journal ID: CMEJ

Canadian Medical Education Journal

EISSN: 1923-1202
Publisher: University of Saskatchewan

ARTICLE INFORMATION
==============================
Subjects: Oc - 4 – 2

Social Support Processes of Students and Clinicians with Disabilities in Healthcare Educational Journeys

Mayer Yael University of British Columbia

Tal Jarus University of British Columbia

Shalev Michal University of British Columbia

Battalova Alfiya University of British Columbia

Yvonne Bulk Laura University of British Columbia

Nimmon Laura University of British Columbia

Lee Michael University of British Columbia

Electronic publication date: 2019 Apr 3

JOURNAL INFORMATION
==============================
NLM Title Abbreviation: Can Med Educ J
Journal ID: Can Med Educ J
NLM Journal ID: 101560935
Journal ID: CMEJ

Canadian Medical Education Journal

EISSN: 1923-1202
Publisher: University of Saskatchewan

ARTICLE INFORMATION
==============================
Subjects: Oc - 4 – 3

Mapping the Health Advocate role across postgraduate medical education

Endres Kaitlin University of Ottawa

Karol Dalia University of Ottawa

Weiman Daniel University of Ottawa

Cowley Lindsay University of Ottawa

Burm Sarah Western University

Dudek Nancy University of Ottawa

LaDonna Kori University of Ottawa

Electronic publication date: 2019 Apr 3

JOURNAL INFORMATION
==============================
NLM Title Abbreviation: Can Med Educ J
Journal ID: Can Med Educ J
NLM Journal ID: 101560935
Journal ID: CMEJ

Canadian Medical Education Journal

EISSN: 1923-1202
Publisher: University of Saskatchewan

ARTICLE INFORMATION
==============================
Subjects: Oc - 4 – 4

Realistic Preparation for Going Global: Pre-departure Training Driven by Role-Playing Case Study Simulations

Sheahan Guy Queen’s University

Carpenter Jennifer Queen’s University

Matzinger Elizabeth Queen’s University

Chan Linda Queen’s University

Soleas Eleftherios Queen’s University

De Sousa Mikaila Queen’s University

Baumhour Jessica Queen’s University

Electronic publication date: 2019 Apr 3

JOURNAL INFORMATION
==============================
NLM Title Abbreviation: Can Med Educ J
Journal ID: Can Med Educ J
NLM Journal ID: 101560935
Journal ID: CMEJ

Canadian Medical Education Journal

EISSN: 1923-1202
Publisher: University of Saskatchewan

ARTICLE INFORMATION
==============================
Subjects: Oc - 4 – 5

Rehearsing the role of 'Health advocate' through research-based theatre: Lessons learned from engaging with lived experiences of homelessness

Hossain Rahat McMaster University

Ramsay Natalie McMaster University

Moore Mo McMaster University

Milo Michael McMaster University

Electronic publication date: 2019 Apr 3

JOURNAL INFORMATION
==============================
NLM Title Abbreviation: Can Med Educ J
Journal ID: Can Med Educ J
NLM Journal ID: 101560935
Journal ID: CMEJ

Canadian Medical Education Journal

EISSN: 1923-1202
Publisher: University of Saskatchewan

ARTICLE INFORMATION
==============================
Subjects: Oc - 4 – 6

Voices from Within: An analysis of physicians' reflective narratives about flaws with the "system"

Moniz Tracy Department of Communication Studies, Mount Saint Vincent University (primary appointment); Faculty of Medicine, Dalhousie University (adjunct)

Pack Rachael Western University

Lingard Lorelei Western University

Watling Chris Western University

Electronic publication date: 2019 Apr 3

JOURNAL INFORMATION
==============================
NLM Title Abbreviation: Can Med Educ J
Journal ID: Can Med Educ J
NLM Journal ID: 101560935
Journal ID: CMEJ

Canadian Medical Education Journal

EISSN: 1923-1202
Publisher: University of Saskatchewan

ARTICLE INFORMATION
==============================
Subjects: Oc - 5 – 1

Simulation through research-based theatre: Using drama in interprofessional education to improve healthcare for persons experiencing homelessness

Gabara Adam Queen’s University

Harrison Rebecca McMaster University

Alaniz Grecia McMaster University

Birk Tanisha McMaster University

Essah Karen McMaster University

Ross Caitlin McMaster University

Hossain Rahat McMaster University

Electronic publication date: 2019 Apr 3

JOURNAL INFORMATION
==============================
NLM Title Abbreviation: Can Med Educ J
Journal ID: Can Med Educ J
NLM Journal ID: 101560935
Journal ID: CMEJ

Canadian Medical Education Journal

EISSN: 1923-1202
Publisher: University of Saskatchewan

ARTICLE INFORMATION
==============================
Subjects: Oc - 5 – 2

Implications of work context on help-seeking and help-provision behaviours in interdisciplinary clinical teams: A multidisciplinary perspective

Kennedy Erin Western University

Lingard Lorelei Western University

Watling Chris Western University

Cristancho Sayra Western University

Parsons Leigh Jeanna Western University

Hernandez-Alejandro Roberto The University of Rochester Medical Center

Electronic publication date: 2019 Apr 3

JOURNAL INFORMATION
==============================
NLM Title Abbreviation: Can Med Educ J
Journal ID: Can Med Educ J
NLM Journal ID: 101560935
Journal ID: CMEJ

Canadian Medical Education Journal

EISSN: 1923-1202
Publisher: University of Saskatchewan

ARTICLE INFORMATION
==============================
Subjects: Oc - 5 – 3

Evidence-based recommendations for collaborative care education programs: A scoping review

Shen Nelson University of Toronto

Sockalingam Sanjeev University of Toronto

Charow Rebecca University Health Network

Bailey Sharon Centre for Addiction and Mental Health

Bernier Thérèse University of Toronto

Freeland Alison University of Toronto

Hawa Aceel University of Toronto

Sur Deepy McMaster University

Wiljer David University of Toronto

Electronic publication date: 2019 Apr 3

JOURNAL INFORMATION
==============================
NLM Title Abbreviation: Can Med Educ J
Journal ID: Can Med Educ J
NLM Journal ID: 101560935
Journal ID: CMEJ

Canadian Medical Education Journal

EISSN: 1923-1202
Publisher: University of Saskatchewan

ARTICLE INFORMATION
==============================
Subjects: Oc - 5 – 4

Using graphic illustrations to uncover how a community of practice can influence the delivery of compassionate healthcare

Dalgarno Nancy Queen’s University

Parsons Trisha Queen’s University

Tregunno Deborah Queen’s University

Flynn Leslie Queen’s University

Joneja Mala Queen’s University

Electronic publication date: 2019 Apr 3

JOURNAL INFORMATION
==============================
NLM Title Abbreviation: Can Med Educ J
Journal ID: Can Med Educ J
NLM Journal ID: 101560935
Journal ID: CMEJ

Canadian Medical Education Journal

EISSN: 1923-1202
Publisher: University of Saskatchewan

ARTICLE INFORMATION
==============================
Subjects: Oc - 5 – 5

Development of an interprofessional trauma-informed care workshop

Pasquale Julia University of Toronto

Kanji Sarah University of Toronto

Marek Rachelle University of Toronto

Toste Aja University of Toronto

Graziano Daniela University of Toronto

Hau Athena University of Toronto

Freeman Sarah University of Toronto

Electronic publication date: 2019 Apr 3

JOURNAL INFORMATION
==============================
NLM Title Abbreviation: Can Med Educ J
Journal ID: Can Med Educ J
NLM Journal ID: 101560935
Journal ID: CMEJ

Canadian Medical Education Journal

EISSN: 1923-1202
Publisher: University of Saskatchewan

ARTICLE INFORMATION
==============================
Subjects: Oc - 5 – 6

Key considerations when sharing interprofessional simulation courses across healthcare contexts

M Naismith Laura University of Toronto

Mylopoulos Maria University of Toronto

Salenieks Therese Centre for Addiction and Mental Health

M Walsh Catharine University of Toronto

Electronic publication date: 2019 Apr 3

JOURNAL INFORMATION
==============================
NLM Title Abbreviation: Can Med Educ J
Journal ID: Can Med Educ J
NLM Journal ID: 101560935
Journal ID: CMEJ

Canadian Medical Education Journal

EISSN: 1923-1202
Publisher: University of Saskatchewan

ARTICLE INFORMATION
==============================
Subjects: Oc - 6 – 1

Monitoring of curriculum implementation- an often neglected process?

Cumyn Annabelle Université de Sherbrooke

Moreau Isabelle Université de Sherbrooke

Samandi Sondos Université de Sherbrooke

Mathieu Sylvie Université de Sherbrooke

Houde Ghislaine Université de Montréal

Gagné Ève-Reine Université de Sherbrooke

Electronic publication date: 2019 Apr 3

JOURNAL INFORMATION
==============================
NLM Title Abbreviation: Can Med Educ J
Journal ID: Can Med Educ J
NLM Journal ID: 101560935
Journal ID: CMEJ

Canadian Medical Education Journal

EISSN: 1923-1202
Publisher: University of Saskatchewan

ARTICLE INFORMATION
==============================
Subjects: Oc - 6 – 2

Humanism in medicine: supporting empathy

Byszewski Anna University of Ottawa

Lochnan Heather University of Ottawa

Forgie Melissa University of Ottawa

Rousseau Philippe University of Ottawa

Electronic publication date: 2019 Apr 3

JOURNAL INFORMATION
==============================
NLM Title Abbreviation: Can Med Educ J
Journal ID: Can Med Educ J
NLM Journal ID: 101560935
Journal ID: CMEJ

Canadian Medical Education Journal

EISSN: 1923-1202
Publisher: University of Saskatchewan

ARTICLE INFORMATION
==============================
Subjects: Oc - 6 – 3

Improving undergraduate instruction in evidence based medicine (EBM): mapping the University of Alberta undergraduate EBM curriculum to national competencies as a tool to facilitate curriculum development.

Goetz Virginia University of Alberta

Do Victor University of Alberta

Hillier Tracey University of Alberta

Electronic publication date: 2019 Apr 3

JOURNAL INFORMATION
==============================
NLM Title Abbreviation: Can Med Educ J
Journal ID: Can Med Educ J
NLM Journal ID: 101560935
Journal ID: CMEJ

Canadian Medical Education Journal

EISSN: 1923-1202
Publisher: University of Saskatchewan

ARTICLE INFORMATION
==============================
Subjects: Oc - 6 – 4

An Innovative Scholar Competency Curriculum in Undergraduate Medical Education

Murray Heather Queen’s University

Jain Kunal Queen’s University

Lee Kevin Queen’s University

Walker Melanie Queen’s University

Electronic publication date: 2019 Apr 3

JOURNAL INFORMATION
==============================
NLM Title Abbreviation: Can Med Educ J
Journal ID: Can Med Educ J
NLM Journal ID: 101560935
Journal ID: CMEJ

Canadian Medical Education Journal

EISSN: 1923-1202
Publisher: University of Saskatchewan

ARTICLE INFORMATION
==============================
Subjects: Oc - 6 – 5

The Opioid Awareness & Support Team (OAST): A novel approach towards education and community engagement at MUN Medicine

Downer Matthew Memorial – University of Newfoundland

Duffley Luke Memorial – University of Newfoundland

Hillier Phil Memorial – University of Newfoundland

Lacey Kieran Memorial – University of Newfoundland

Lewis Madison Memorial – University of Newfoundland

Lehr Josh Memorial – University of Newfoundland

Turner Brooke Memorial – University of Newfoundland

Jill Allison Dr. Memorial – University of Newfoundland

Electronic publication date: 2019 Apr 3

JOURNAL INFORMATION
==============================
NLM Title Abbreviation: Can Med Educ J
Journal ID: Can Med Educ J
NLM Journal ID: 101560935
Journal ID: CMEJ

Canadian Medical Education Journal

EISSN: 1923-1202
Publisher: University of Saskatchewan

ARTICLE INFORMATION
==============================
Subjects: Oc - 6 – 6

Integration of Community-Based Indigenous Health Education into Pre-Clerkship Curriculum

Amer Ali Layla Western University

Hansen Brenna Western University

Poole Cassie Western University

Mandawe Erik Western University

Kim George Western University

Electronic publication date: 2019 Apr 3

JOURNAL INFORMATION
==============================
NLM Title Abbreviation: Can Med Educ J
Journal ID: Can Med Educ J
NLM Journal ID: 101560935
Journal ID: CMEJ

Canadian Medical Education Journal

EISSN: 1923-1202
Publisher: University of Saskatchewan

ARTICLE INFORMATION
==============================
Subjects: Oc - 7 – 1

Near-Peer Interactions and the advice delivered before and after curriculum change.

Huang Kelly University of British Columbia

Mak David University of British Columbia

Eva Kevin University of British Columbia

Hafferty Fred Mayo Clinic

Electronic publication date: 2019 Apr 3

JOURNAL INFORMATION
==============================
NLM Title Abbreviation: Can Med Educ J
Journal ID: Can Med Educ J
NLM Journal ID: 101560935
Journal ID: CMEJ

Canadian Medical Education Journal

EISSN: 1923-1202
Publisher: University of Saskatchewan

ARTICLE INFORMATION
==============================
Subjects: Oc - 7 – 2

Flexible Enhanced Learning: A Model for Student-Defined Undergraduate Medical Education at the University of British Columbia

Lazenby Richard University of British Columbia

Mui Alice University of British Columbia

Cooper Elson S. Floyd Dawn College of Medicine Washington State University

Electronic publication date: 2019 Apr 3

JOURNAL INFORMATION
==============================
NLM Title Abbreviation: Can Med Educ J
Journal ID: Can Med Educ J
NLM Journal ID: 101560935
Journal ID: CMEJ

Canadian Medical Education Journal

EISSN: 1923-1202
Publisher: University of Saskatchewan

ARTICLE INFORMATION
==============================
Subjects: Oc - 7 – 3

Shining a light into the black box of group learning

Park Charles University of British Columbia

Wu Claire University of British Columbia

Regehr Glenn University of British Columbia

Electronic publication date: 2019 Apr 3

JOURNAL INFORMATION
==============================
NLM Title Abbreviation: Can Med Educ J
Journal ID: Can Med Educ J
NLM Journal ID: 101560935
Journal ID: CMEJ

Canadian Medical Education Journal

EISSN: 1923-1202
Publisher: University of Saskatchewan

ARTICLE INFORMATION
==============================
Subjects: Oc - 7 – 4

Evaluation of Feedback on Graded Team Assignments (GTAs) at Queen's University School of Medicine

Belyea Andrew Queen’s University

Gibson Michelle Queen’s University

Katsoulas Eleni Queen’s University

Electronic publication date: 2019 Apr 3

JOURNAL INFORMATION
==============================
NLM Title Abbreviation: Can Med Educ J
Journal ID: Can Med Educ J
NLM Journal ID: 101560935
Journal ID: CMEJ

Canadian Medical Education Journal

EISSN: 1923-1202
Publisher: University of Saskatchewan

ARTICLE INFORMATION
==============================
Subjects: Oc - 7 – 5

Fitting a square peg in a round hole: students as partners in educational governance

Bilodeau Philippe-Antoine McGill

Mei Liu Xin McGill

Cummings Beth-Ann McGill

Electronic publication date: 2019 Apr 3

JOURNAL INFORMATION
==============================
NLM Title Abbreviation: Can Med Educ J
Journal ID: Can Med Educ J
NLM Journal ID: 101560935
Journal ID: CMEJ

Canadian Medical Education Journal

EISSN: 1923-1202
Publisher: University of Saskatchewan

ARTICLE INFORMATION
==============================
Subjects: Oc - 7 – 6

Evaluating the Impact of Reducing Clerkship Form Length and Nudging Face-to-Face Assessments on Validity

Daniels Vijay University of Alberta

Lai Hollis University of Alberta

Forbes Karen University of Alberta

Hillier Tracey University of Alberta

Electronic publication date: 2019 Apr 3

JOURNAL INFORMATION
==============================
NLM Title Abbreviation: Can Med Educ J
Journal ID: Can Med Educ J
NLM Journal ID: 101560935
Journal ID: CMEJ

Canadian Medical Education Journal

EISSN: 1923-1202
Publisher: University of Saskatchewan

ARTICLE INFORMATION
==============================
Subjects: Oc - 8 – 1

Preceptor Modelling of Wellness and Student Perceptions

Galbraith Lauren University of British Columbia

Grisdale Mackenzie McMaster University

Hutchison Carol University of Calgary

Paget Mike University of Calgary

Electronic publication date: 2019 Apr 3

JOURNAL INFORMATION
==============================
NLM Title Abbreviation: Can Med Educ J
Journal ID: Can Med Educ J
NLM Journal ID: 101560935
Journal ID: CMEJ

Canadian Medical Education Journal

EISSN: 1923-1202
Publisher: University of Saskatchewan

ARTICLE INFORMATION
==============================
Subjects: Oc - 8 – 2

Acting on What we Already Know: Supporting Resiliency Among Resident Physicians in Canada

Ritsma Amanda McMaster University

Leekha Aarun Memorial – University of Newfoundland

Electronic publication date: 2019 Apr 3

JOURNAL INFORMATION
==============================
NLM Title Abbreviation: Can Med Educ J
Journal ID: Can Med Educ J
NLM Journal ID: 101560935
Journal ID: CMEJ

Canadian Medical Education Journal

EISSN: 1923-1202
Publisher: University of Saskatchewan

ARTICLE INFORMATION
==============================
Subjects: Oc - 8 – 3

Performing Under Pressure: Stress in Surgical Practice

McQueen Sydney University of Toronto

Hammond Melanie Mobilio University of Toronto

Shehata Adam University of Toronto

Moulton Carol-Anne University of Toronto

Electronic publication date: 2019 Apr 3

JOURNAL INFORMATION
==============================
NLM Title Abbreviation: Can Med Educ J
Journal ID: Can Med Educ J
NLM Journal ID: 101560935
Journal ID: CMEJ

Canadian Medical Education Journal

EISSN: 1923-1202
Publisher: University of Saskatchewan

ARTICLE INFORMATION
==============================
Subjects: Oc - 8 – 4

Proposed National Standards for Resident Call Rooms

Wynick Avery University of Alberta

Zuo Kevin University of Toronto

Bigham Blair McMaster University

Morrow Justyne University of Calgary

Ong Melody University of Manitoba

Electronic publication date: 2019 Apr 3

JOURNAL INFORMATION
==============================
NLM Title Abbreviation: Can Med Educ J
Journal ID: Can Med Educ J
NLM Journal ID: 101560935
Journal ID: CMEJ

Canadian Medical Education Journal

EISSN: 1923-1202
Publisher: University of Saskatchewan

ARTICLE INFORMATION
==============================
Subjects: Oc - 8 – 5

The stressed heart: using simulation to assess mobile measures of heart rate variability

LeBlanc Vicki University of Ottawa

Mastoras George University of Ottawa

Hicks Christopher University of Toronto

Aucoin Philip University of Ottawa

O'Rielly Connor University of Calgary

Electronic publication date: 2019 Apr 3

JOURNAL INFORMATION
==============================
NLM Title Abbreviation: Can Med Educ J
Journal ID: Can Med Educ J
NLM Journal ID: 101560935
Journal ID: CMEJ

Canadian Medical Education Journal

EISSN: 1923-1202
Publisher: University of Saskatchewan

ARTICLE INFORMATION
==============================
Subjects: Oc - 8 – 6

Choosing wisely: An autoethnographic exploration of self-preservation in clerkship

Coret Alon University of Toronto

Perrella Andrew University of Toronto, University of Toronto

Regehr Glenn University of British Columbia

Farrell Laura University of British Columbia

Electronic publication date: 2019 Apr 3

JOURNAL INFORMATION
==============================
NLM Title Abbreviation: Can Med Educ J
Journal ID: Can Med Educ J
NLM Journal ID: 101560935
Journal ID: CMEJ

Canadian Medical Education Journal

EISSN: 1923-1202
Publisher: University of Saskatchewan

ARTICLE INFORMATION
==============================
Subjects: Od - 1 – 1

Learning analytics play a role in competency-based CPD: The case of an ECG interpretation course participants' learning data

Burnett Chloe University of Calgary

Weeks Sarah University of Calgary

Ryan Caitlin University of Calgary

Dongo Tendai University of Calgary

Burak Kelly University of Calgary

Electronic publication date: 2019 Apr 3

JOURNAL INFORMATION
==============================
NLM Title Abbreviation: Can Med Educ J
Journal ID: Can Med Educ J
NLM Journal ID: 101560935
Journal ID: CMEJ

Canadian Medical Education Journal

EISSN: 1923-1202
Publisher: University of Saskatchewan

ARTICLE INFORMATION
==============================
Subjects: Od - 1 – 2

Understanding the relationship between data-informed learning and orientation to lifelong learning

Sockalingam Sanjeev University of Toronto

Wiljer David University of Toronto

Davis Dave Mohammed Bin Rashid University

Giuliani Meredith University of Toronto

Okrainec Allan University of Toronto

Silver Ivan University of Toronto

Campbell Craig The Royal College of Physicians and Surgeons

Mylopoulos Maria University of Toronto

Tavares Walter University of Toronto

Charow Rebecca University Health Network

Electronic publication date: 2019 Apr 3

JOURNAL INFORMATION
==============================
NLM Title Abbreviation: Can Med Educ J
Journal ID: Can Med Educ J
NLM Journal ID: 101560935
Journal ID: CMEJ

Canadian Medical Education Journal

EISSN: 1923-1202
Publisher: University of Saskatchewan

ARTICLE INFORMATION
==============================
Subjects: Od - 1 – 3

Role of Project ECHO in quality improvement: data from mental health and addictions

Sockalingam Sanjeev University of Toronto

Kirvan Anne Centre for Addiction and Mental Health

Pereira Cheryl Centre for Addiction and Mental Health

Rajaratnam Thiyake Centre for Addiction and Mental Health

El-Zein Yasmeenah Centre for Addiction and Mental Health

Serhal Eva Centre for Addiction and Mental Health

Crawford Allison University of Toronto

Electronic publication date: 2019 Apr 3

JOURNAL INFORMATION
==============================
NLM Title Abbreviation: Can Med Educ J
Journal ID: Can Med Educ J
NLM Journal ID: 101560935
Journal ID: CMEJ

Canadian Medical Education Journal

EISSN: 1923-1202
Publisher: University of Saskatchewan

ARTICLE INFORMATION
==============================
Subjects: Od - 1 – 4

Specialist by day, Generalist by night - A needs assessment approach for Anesthesiology

Brock Michael Western University

Schulz Valerie Western University

Dixon David Western University

Electronic publication date: 2019 Apr 3

JOURNAL INFORMATION
==============================
NLM Title Abbreviation: Can Med Educ J
Journal ID: Can Med Educ J
NLM Journal ID: 101560935
Journal ID: CMEJ

Canadian Medical Education Journal

EISSN: 1923-1202
Publisher: University of Saskatchewan

ARTICLE INFORMATION
==============================
Subjects: Od - 1 – 5

Wound Care Continuing Professional Development and Bootcamps: Interprofessional before it was cool

Sibbald Gary University of Toronto

Smith Karen Queen’s University

McDiarmid Laura Queen’s University

Deacon James Queen’s University

Heil Jolene Queen’s University

Mangan Cynthia Queen’s University

Soleas Eleftherios Queen’s University

Electronic publication date: 2019 Apr 3

JOURNAL INFORMATION
==============================
NLM Title Abbreviation: Can Med Educ J
Journal ID: Can Med Educ J
NLM Journal ID: 101560935
Journal ID: CMEJ

Canadian Medical Education Journal

EISSN: 1923-1202
Publisher: University of Saskatchewan

ARTICLE INFORMATION
==============================
Subjects: Od - 2 – 1

Impact of a Student-Organized 12-Hour Indigenous Health Elective on Student Learning Outcomes

Homer Kai University of Alberta

Lebreton Michelle University of Alberta

Kassam Shehzad University of Alberta

Deutscher Julianna University of Alberta

Christensen Jeremy University of Alberta

Mildenberger Adam University of Alberta

Mensik Nicole University of Alberta

Davidson Riley University of Alberta

Konkin Jill University of Alberta

Electronic publication date: 2019 Apr 3

JOURNAL INFORMATION
==============================
NLM Title Abbreviation: Can Med Educ J
Journal ID: Can Med Educ J
NLM Journal ID: 101560935
Journal ID: CMEJ

Canadian Medical Education Journal

EISSN: 1923-1202
Publisher: University of Saskatchewan

ARTICLE INFORMATION
==============================
Subjects: Od - 2 – 2

Effect of Detailed OSCE Score Reporting on Student Learning

Ortiz Silvia University of Alberta

Daniels Vijay University of Alberta

Lai Hollis University of Alberta

Yoon Minn University of Alberta

Bulut Okan University of Alberta

Hillier Tracey University of Alberta

Electronic publication date: 2019 Apr 3

JOURNAL INFORMATION
==============================
NLM Title Abbreviation: Can Med Educ J
Journal ID: Can Med Educ J
NLM Journal ID: 101560935
Journal ID: CMEJ

Canadian Medical Education Journal

EISSN: 1923-1202
Publisher: University of Saskatchewan

ARTICLE INFORMATION
==============================
Subjects: Od - 2 – 3

Following medical doodlers on Instagram: A good med student learning strategy?

Cox Susan University of British Columbia

Ann Courneya Carol University of British Columbia

Electronic publication date: 2019 Apr 3

JOURNAL INFORMATION
==============================
NLM Title Abbreviation: Can Med Educ J
Journal ID: Can Med Educ J
NLM Journal ID: 101560935
Journal ID: CMEJ

Canadian Medical Education Journal

EISSN: 1923-1202
Publisher: University of Saskatchewan

ARTICLE INFORMATION
==============================
Subjects: Od - 2 – 4

The effectiveness of different models for observation-based learning

Kalun Portia McMaster University

Kalun Portia McMaster University

Zering Jennifer McMaster University

Cyfko John McMaster University

Sideris Beth McMaster University

Sonnadara Ranil McMaster University

Electronic publication date: 2019 Apr 3

JOURNAL INFORMATION
==============================
NLM Title Abbreviation: Can Med Educ J
Journal ID: Can Med Educ J
NLM Journal ID: 101560935
Journal ID: CMEJ

Canadian Medical Education Journal

EISSN: 1923-1202
Publisher: University of Saskatchewan

ARTICLE INFORMATION
==============================
Subjects: Od - 2 – 5

Liminality in co-produced health professions education: A phenomenological study

Kalocsai Csilla Centre for Addiction and Mental Health

Agrawal Sacha University of Toronto

Kalocsai Csilla Centre for Addiction and Mental Health

Capponi Pat University of Toronto

Kidd Sean University of Toronto

Wiljer David University of Toronto

Ringsted Charlotte Aarhus University

Soklaridis Sophie University of Toronto

Electronic publication date: 2019 Apr 3

JOURNAL INFORMATION
==============================
NLM Title Abbreviation: Can Med Educ J
Journal ID: Can Med Educ J
NLM Journal ID: 101560935
Journal ID: CMEJ

Canadian Medical Education Journal

EISSN: 1923-1202
Publisher: University of Saskatchewan

ARTICLE INFORMATION
==============================
Subjects: Od - 2 – 6

Creating an eLearning module for Faculty Development self-study.

Bordman Risa University of Toronto

Mayhew Linda McMaster University

Shahid Aiman McMaster University

Electronic publication date: 2019 Apr 3

JOURNAL INFORMATION
==============================
NLM Title Abbreviation: Can Med Educ J
Journal ID: Can Med Educ J
NLM Journal ID: 101560935
Journal ID: CMEJ

Canadian Medical Education Journal

EISSN: 1923-1202
Publisher: University of Saskatchewan

ARTICLE INFORMATION
==============================
Subjects: Od - 3 – 1

The Medicolegal Risks of Clinical Supervision: A 60-Year Review

Boileau Elisabeth Université de Sherbrooke

St-Onge Christina Université de Sherbrooke

Electronic publication date: 2019 Apr 3

JOURNAL INFORMATION
==============================
NLM Title Abbreviation: Can Med Educ J
Journal ID: Can Med Educ J
NLM Journal ID: 101560935
Journal ID: CMEJ

Canadian Medical Education Journal

EISSN: 1923-1202
Publisher: University of Saskatchewan

ARTICLE INFORMATION
==============================
Subjects: Od - 3 – 2

Making sense of our own mistakes: a critical narrative analysis of experienced generalist physician stories of error

Kandasamy Sujane McMaster University

Monteiro Sandra McMaster University

Vanstone Meredith McMaster University

Colvin Eamon University of Ottawa

Chan Teresa McMaster University

Sherbino Jonathan McMaster University

Electronic publication date: 2019 Apr 3

JOURNAL INFORMATION
==============================
NLM Title Abbreviation: Can Med Educ J
Journal ID: Can Med Educ J
NLM Journal ID: 101560935
Journal ID: CMEJ

Canadian Medical Education Journal

EISSN: 1923-1202
Publisher: University of Saskatchewan

ARTICLE INFORMATION
==============================
Subjects: Od - 3 – 3

Medicolegal Education - Are Physicians Adequately Trained in Law?

Pangli Harpreet University of British Columbia

Tai Teresa University of British Columbia

Parhar Gurdeep University of British Columbia

Electronic publication date: 2019 Apr 3

JOURNAL INFORMATION
==============================
NLM Title Abbreviation: Can Med Educ J
Journal ID: Can Med Educ J
NLM Journal ID: 101560935
Journal ID: CMEJ

Canadian Medical Education Journal

EISSN: 1923-1202
Publisher: University of Saskatchewan

ARTICLE INFORMATION
==============================
Subjects: Od - 3 – 4

The civil legal experience of Canadian trainees

McDougall Allan Canadian Medical Protective Association

Mojaverian Nassim Canadian Medical Protective Association

Steen Anne Canadian Medical Protective Association

Yang Qian Canadian Medical Protective Association

Nuth Janet Canadian Medical Protective Association

Tsai Ellen Canadian Medical Protective Association

Lee Shirley Canadian Medical Protective Association

Lefebvre Guylaine Canadian Medical Protective Association

Calder Lisa Canadian Medical Protective Association

Electronic publication date: 2019 Apr 3

JOURNAL INFORMATION
==============================
NLM Title Abbreviation: Can Med Educ J
Journal ID: Can Med Educ J
NLM Journal ID: 101560935
Journal ID: CMEJ

Canadian Medical Education Journal

EISSN: 1923-1202
Publisher: University of Saskatchewan

ARTICLE INFORMATION
==============================
Subjects: Od - 3 – 5

Epidemiology of competence: the theory and practice of risks and supports to competence

Glover Susan Takahashi University of Toronto

Nayer Marla University of Toronto

Electronic publication date: 2019 Apr 3

JOURNAL INFORMATION
==============================
NLM Title Abbreviation: Can Med Educ J
Journal ID: Can Med Educ J
NLM Journal ID: 101560935
Journal ID: CMEJ

Canadian Medical Education Journal

EISSN: 1923-1202
Publisher: University of Saskatchewan

ARTICLE INFORMATION
==============================
Subjects: Od - 3 – 6

'What…you can't tell left from right? Medical students experiences in making laterality decisions

Gormley Gerry Queen's University Belfast

Brennan Carl Queen's University Belfast

Demspter Martin Queen's University Belfast

Electronic publication date: 2019 Apr 3

JOURNAL INFORMATION
==============================
NLM Title Abbreviation: Can Med Educ J
Journal ID: Can Med Educ J
NLM Journal ID: 101560935
Journal ID: CMEJ

Canadian Medical Education Journal

EISSN: 1923-1202
Publisher: University of Saskatchewan

ARTICLE INFORMATION
==============================
Subjects: Od - 4 – 1

Task Demands in OSCEs Influence Learning Strategies and Diagnostic Reasoning

Lafleur Alexandre Université Laval

Laflamme Jonathan Université Laval

Leppink Jimmie Maastricht University

Côté Luc Université Laval

Electronic publication date: 2019 Apr 3

JOURNAL INFORMATION
==============================
NLM Title Abbreviation: Can Med Educ J
Journal ID: Can Med Educ J
NLM Journal ID: 101560935
Journal ID: CMEJ

Canadian Medical Education Journal

EISSN: 1923-1202
Publisher: University of Saskatchewan

ARTICLE INFORMATION
==============================
Subjects: Od - 4 – 2

Unpicking the dominance of standardisation in OSCEs using Institutional Ethnography

P Kearney Grainne Queen's University, Belfast

Johnston Jenny Queen's University, Belfast

D Hart Nigel Queen's University, Belfast

Gormley Gerry Queen's University, Belfast

Electronic publication date: 2019 Apr 3

JOURNAL INFORMATION
==============================
NLM Title Abbreviation: Can Med Educ J
Journal ID: Can Med Educ J
NLM Journal ID: 101560935
Journal ID: CMEJ

Canadian Medical Education Journal

EISSN: 1923-1202
Publisher: University of Saskatchewan

ARTICLE INFORMATION
==============================
Subjects: Od - 4 – 3

Global rating of OSCE performance is a better predictor of entrustment ratings than checklist scores

Desy Janeve University of Calgary

Coderre Sylvain University of Calgary

Ma Irene University of Calgary

Veale Pamela University of Calgary

Paget Mike University of Calgary

Busche Kevin University of Calgary

McLaughlin Kevin University of Calgary

Electronic publication date: 2019 Apr 3

JOURNAL INFORMATION
==============================
NLM Title Abbreviation: Can Med Educ J
Journal ID: Can Med Educ J
NLM Journal ID: 101560935
Journal ID: CMEJ

Canadian Medical Education Journal

EISSN: 1923-1202
Publisher: University of Saskatchewan

ARTICLE INFORMATION
==============================
Subjects: Od - 4 – 4

OSCEs Unplugged: critical exploration of stakeholder perspectives

Reid Helen Queen's University, Belfast

Johnston Jennifer Queen's University, Belfast

Gormley Gerard Queen's University, Belfast

Dornan Tim Queen's University, Belfast

Corrigan Mairead Queen's University, Belfast

Cantillon Peter National University of Ireland, Galway

Electronic publication date: 2019 Apr 3

JOURNAL INFORMATION
==============================
NLM Title Abbreviation: Can Med Educ J
Journal ID: Can Med Educ J
NLM Journal ID: 101560935
Journal ID: CMEJ

Canadian Medical Education Journal

EISSN: 1923-1202
Publisher: University of Saskatchewan

ARTICLE INFORMATION
==============================
Subjects: Od - 4 – 5

Insight into retake performances on 12 high-stake OSCE stations to help guide policy decisions

Coetzee Karen Touchstone Institute

Monteiro Sandra McMaster University

Electronic publication date: 2019 Apr 3

JOURNAL INFORMATION
==============================
NLM Title Abbreviation: Can Med Educ J
Journal ID: Can Med Educ J
NLM Journal ID: 101560935
Journal ID: CMEJ

Canadian Medical Education Journal

EISSN: 1923-1202
Publisher: University of Saskatchewan

ARTICLE INFORMATION
==============================
Subjects: Od - 4 – 6

Comment un ouvrage pédagogique médical écrit par et pour les étudiants a changé la manière de se préparer pour les ECOS

Richard Élizabeth Université Laval

Ménard-Cholette Vincent Université Laval

Thériault Julie Université Laval

Wang Emily Université Laval

Electronic publication date: 2019 Apr 3

JOURNAL INFORMATION
==============================
NLM Title Abbreviation: Can Med Educ J
Journal ID: Can Med Educ J
NLM Journal ID: 101560935
Journal ID: CMEJ

Canadian Medical Education Journal

EISSN: 1923-1202
Publisher: University of Saskatchewan

ARTICLE INFORMATION
==============================
Subjects: Od - 5 – 1

The flipped academic half day program in Physical Medicine and Rehabilitation: Residents' perceptions about what works

Askari Sussan Queen’s University

Dalgarno Nancy Queen’s University

Langshaw Fredrick Queen’s University

Siqueira Izabelle Queen’s University

Egan Rylan Queen’s University

Trier Jessica Queen’s University

Electronic publication date: 2019 Apr 3

JOURNAL INFORMATION
==============================
NLM Title Abbreviation: Can Med Educ J
Journal ID: Can Med Educ J
NLM Journal ID: 101560935
Journal ID: CMEJ

Canadian Medical Education Journal

EISSN: 1923-1202
Publisher: University of Saskatchewan

ARTICLE INFORMATION
==============================
Subjects: Od - 5 – 2

Modelling for Physician Planning in Ontario - Keeping it Simple and Making it Relevant. Part 1 (The Story of Supply and Activity)

Abrahams Caroline University of Toronto

Bandiera Glen University of Toronto

Babcock Glenys University of Toronto

Shaikhlislamova Natasha University of Toronto

Carter Michael University of Toronto

Strum Scott University of Toronto

Warren Blair University of Toronto

Electronic publication date: 2019 Apr 3

JOURNAL INFORMATION
==============================
NLM Title Abbreviation: Can Med Educ J
Journal ID: Can Med Educ J
NLM Journal ID: 101560935
Journal ID: CMEJ

Canadian Medical Education Journal

EISSN: 1923-1202
Publisher: University of Saskatchewan

ARTICLE INFORMATION
==============================
Subjects: Od - 5 – 3

Strengths and paradoxes in medical leadership education: can postgraduate programs meet contemporary demands for health systems leadership?

Tannenbaum David University of Toronto

Onyura Betty University of Toronto

Freeman Risa University of Toronto

Crann Sara University of Windsor

Kay Whittaker Mary University of Toronto

Murdoch Stuart University of Toronto

Electronic publication date: 2019 Apr 3

JOURNAL INFORMATION
==============================
NLM Title Abbreviation: Can Med Educ J
Journal ID: Can Med Educ J
NLM Journal ID: 101560935
Journal ID: CMEJ

Canadian Medical Education Journal

EISSN: 1923-1202
Publisher: University of Saskatchewan

ARTICLE INFORMATION
==============================
Subjects: Od - 5 – 4

Effect of Resident Fatigue on Driving

Kent Vincent Le Northern Ontario School of Medicine

Maxwell Hillary Lakehead University

Bedard Michel Lakehead University

DeBakker Peter Northern Ontario School of Medicine

Chan Derrick University of British Columbia

Weaver Bruce Lakehead University

Electronic publication date: 2019 Apr 3

JOURNAL INFORMATION
==============================
NLM Title Abbreviation: Can Med Educ J
Journal ID: Can Med Educ J
NLM Journal ID: 101560935
Journal ID: CMEJ

Canadian Medical Education Journal

EISSN: 1923-1202
Publisher: University of Saskatchewan

ARTICLE INFORMATION
==============================
Subjects: Od - 5 – 5

Anticipation or Avoidance: Internal Medicine Resident Experiences Performing Invasive Bedside Procedures

Louis Alyssa University of Toronto

Lee Christie University of Toronto

Ginsburg Shiphra University of Toronto

Page Andrea University of Toronto

Electronic publication date: 2019 Apr 3

JOURNAL INFORMATION
==============================
NLM Title Abbreviation: Can Med Educ J
Journal ID: Can Med Educ J
NLM Journal ID: 101560935
Journal ID: CMEJ

Canadian Medical Education Journal

EISSN: 1923-1202
Publisher: University of Saskatchewan

ARTICLE INFORMATION
==============================
Subjects: Od - 5 – 6

Implementing Competency Based Medical Education in PGME - A Three Year Longitudinal Study

Dagnone Damon Queen’s University

Stockley Denise Queen’s University

Hussain Alicia Queen’s University

Hastings-Truelove Amber Queen’s University

Electronic publication date: 2019 Apr 3

JOURNAL INFORMATION
==============================
NLM Title Abbreviation: Can Med Educ J
Journal ID: Can Med Educ J
NLM Journal ID: 101560935
Journal ID: CMEJ

Canadian Medical Education Journal

EISSN: 1923-1202
Publisher: University of Saskatchewan

ARTICLE INFORMATION
==============================
Subjects: Od - 6 – 1

Roadmap for Artificial Intelligence-enhanced Assessment in Competency-Based Medical Education

Jurado-Nunez Alma McMaster University

Chan Teresa McMaster University

Electronic publication date: 2019 Apr 3

JOURNAL INFORMATION
==============================
NLM Title Abbreviation: Can Med Educ J
Journal ID: Can Med Educ J
NLM Journal ID: 101560935
Journal ID: CMEJ

Canadian Medical Education Journal

EISSN: 1923-1202
Publisher: University of Saskatchewan

ARTICLE INFORMATION
==============================
Subjects: Od - 6 – 2

Using electronic health record data to assess emergency medicine trainees' independent and interdependent performance

Sebok-Syer Stefanie Stanford University

Shepherd Lisa Western University

Dukelow Adam Western University

Pack Rachel Western University

McConnell Allison Western University

Sedran Robert Western University

Lingard Lorelei Western University

Electronic publication date: 2019 Apr 3

JOURNAL INFORMATION
==============================
NLM Title Abbreviation: Can Med Educ J
Journal ID: Can Med Educ J
NLM Journal ID: 101560935
Journal ID: CMEJ

Canadian Medical Education Journal

EISSN: 1923-1202
Publisher: University of Saskatchewan

ARTICLE INFORMATION
==============================
Subjects: Od - 6 – 3

Validity as a social imperative: perceptions from individuals involved in assessment

Marceau Mélanie Université de Sherbrooke

St-Onge Christina Université de Sherbrooke

Gallagher Frances Université de Sherbrooke

Young Meredith McGill

Electronic publication date: 2019 Apr 3

JOURNAL INFORMATION
==============================
NLM Title Abbreviation: Can Med Educ J
Journal ID: Can Med Educ J
NLM Journal ID: 101560935
Journal ID: CMEJ

Canadian Medical Education Journal

EISSN: 1923-1202
Publisher: University of Saskatchewan

ARTICLE INFORMATION
==============================
Subjects: Od - 6 – 4

Assessment of Collaborator Competencies: Understanding the Landscape in Undergraduate Medicine

Newton Christie University of British Columbia

Langlois Sylvia University of Toronto

Electronic publication date: 2019 Apr 3

JOURNAL INFORMATION
==============================
NLM Title Abbreviation: Can Med Educ J
Journal ID: Can Med Educ J
NLM Journal ID: 101560935
Journal ID: CMEJ

Canadian Medical Education Journal

EISSN: 1923-1202
Publisher: University of Saskatchewan

ARTICLE INFORMATION
==============================
Subjects: Od - 6 – 5

Learner and Clinician Interpretation of Collaboration in Interprofessional Competency Assessment

Langlois Sylvia University of Toronto

Brijmohan Amanda University of Toronto

Vardy Graham University of Toronto

Stirling Ashley University of Toronto

Kanofsky Sharona University of Toronto

Paulenko Tracy University Health Network

Lee Annie University of Toronto

Electronic publication date: 2019 Apr 3

JOURNAL INFORMATION
==============================
NLM Title Abbreviation: Can Med Educ J
Journal ID: Can Med Educ J
NLM Journal ID: 101560935
Journal ID: CMEJ

Canadian Medical Education Journal

EISSN: 1923-1202
Publisher: University of Saskatchewan

ARTICLE INFORMATION
==============================
Subjects: Od - 6 – 6

Borderline group standard setting: Four approaches for small scale OSCEs

Coetzee Karen Touchstone Institute

Smee Sydney Medical Council of Canada

Bartman Ilona Medical Council of Canada

Sebok-Syer Stefanie Stanford University

Roy Marguerite Medical Council of Canada

Monteiro Sandra McMaster University

Electronic publication date: 2019 Apr 3

JOURNAL INFORMATION
==============================
NLM Title Abbreviation: Can Med Educ J
Journal ID: Can Med Educ J
NLM Journal ID: 101560935
Journal ID: CMEJ

Canadian Medical Education Journal

EISSN: 1923-1202
Publisher: University of Saskatchewan

ARTICLE INFORMATION
==============================
Subjects: Od - 7 – 1

Evaluation of Curricular Segment Contribution to Educational Efficacy: Development of a Program Efficacy Review Process

Trinder Krista University of Saskatchewan

Taylor-Gjevre Regina University of Saskatchewan

Electronic publication date: 2019 Apr 3

JOURNAL INFORMATION
==============================
NLM Title Abbreviation: Can Med Educ J
Journal ID: Can Med Educ J
NLM Journal ID: 101560935
Journal ID: CMEJ

Canadian Medical Education Journal

EISSN: 1923-1202
Publisher: University of Saskatchewan

ARTICLE INFORMATION
==============================
Subjects: Od - 7 – 2

Medical Histology without Live Lectures: A Pilot Study of Online Learning

Nichol Helen University of Saskatchewan

Gilmer Susan University of Saskatchewan

Trinder Krista University of Saskatchewan

Electronic publication date: 2019 Apr 3

JOURNAL INFORMATION
==============================
NLM Title Abbreviation: Can Med Educ J
Journal ID: Can Med Educ J
NLM Journal ID: 101560935
Journal ID: CMEJ

Canadian Medical Education Journal

EISSN: 1923-1202
Publisher: University of Saskatchewan

ARTICLE INFORMATION
==============================
Subjects: Od - 7 – 3

The Role of the Health Professional Educator in Undergraduate Medical Student Education: A Formal Curriculum

Young Sherylan University of Toronto

Goncz Andrea University of Toronto

Chong Evan University of Toronto

Electronic publication date: 2019 Apr 3

JOURNAL INFORMATION
==============================
NLM Title Abbreviation: Can Med Educ J
Journal ID: Can Med Educ J
NLM Journal ID: 101560935
Journal ID: CMEJ

Canadian Medical Education Journal

EISSN: 1923-1202
Publisher: University of Saskatchewan

ARTICLE INFORMATION
==============================
Subjects: Od - 7 – 4

Curricular and extracurricular interprofessional learning opportunities for medical students: an evaluation

Cheon Stephanie Queen’s University

Sadacharam Darsan Queen’s University

Davidson Lindsay Queen’s University

Electronic publication date: 2019 Apr 3

JOURNAL INFORMATION
==============================
NLM Title Abbreviation: Can Med Educ J
Journal ID: Can Med Educ J
NLM Journal ID: 101560935
Journal ID: CMEJ

Canadian Medical Education Journal

EISSN: 1923-1202
Publisher: University of Saskatchewan

ARTICLE INFORMATION
==============================
Subjects: Od - 7 – 5

Promoting medical student career decisions: The Pre-clerkship Residency Exploration Program (PREP), a structured elective program

Dow Todd Dalhousie University

Haupt Sebastian Dalhousie University

Smyth Mike Dalhousie University

Thomas Toguri J Dalhousie University

Raju Kavita Dalhousie University

Roberts Alysha Dalhousie University

MacLeod Anna Dalhousie University

Electronic publication date: 2019 Apr 3

JOURNAL INFORMATION
==============================
NLM Title Abbreviation: Can Med Educ J
Journal ID: Can Med Educ J
NLM Journal ID: 101560935
Journal ID: CMEJ

Canadian Medical Education Journal

EISSN: 1923-1202
Publisher: University of Saskatchewan

ARTICLE INFORMATION
==============================
Subjects: Od - 7 – 6

Medical students' perspectives of the impact of pimping on their learning experience using a self-determination theory framework

Palmer Ashley University of Saskatchewan

Malin Greg University of Saskatchewan

Electronic publication date: 2019 Apr 3

JOURNAL INFORMATION
==============================
NLM Title Abbreviation: Can Med Educ J
Journal ID: Can Med Educ J
NLM Journal ID: 101560935
Journal ID: CMEJ

Canadian Medical Education Journal

EISSN: 1923-1202
Publisher: University of Saskatchewan

ARTICLE INFORMATION
==============================
Subjects: Od - 8 – 1

Exploring physicians' resistance to resilience training

LaDonna Kori University of Ottawa

Cowley Lindsay University of Ottawa

Touchie Claire University of Ottawa

LeBlanc Vicki University of Ottawa, Philip Wells University of Ottawa

Spilg Edward University of Ottawa

Electronic publication date: 2019 Apr 3

JOURNAL INFORMATION
==============================
NLM Title Abbreviation: Can Med Educ J
Journal ID: Can Med Educ J
NLM Journal ID: 101560935
Journal ID: CMEJ

Canadian Medical Education Journal

EISSN: 1923-1202
Publisher: University of Saskatchewan

ARTICLE INFORMATION
==============================
Subjects: Od - 8 – 2

'Resilience' in its many forms: who is responsible for medical trainees wellness?

Muhammad Taaha University of Toronto

Khan Rabia University of Toronto

Athina (Tina) Martimianakis Maria University of Toronto

Paul Robert University of Toronto

Electronic publication date: 2019 Apr 3

JOURNAL INFORMATION
==============================
NLM Title Abbreviation: Can Med Educ J
Journal ID: Can Med Educ J
NLM Journal ID: 101560935
Journal ID: CMEJ

Canadian Medical Education Journal

EISSN: 1923-1202
Publisher: University of Saskatchewan

ARTICLE INFORMATION
==============================
Subjects: Od - 8 – 3

Evaluating Student Perceptions of the Resilience Curriculum at the MD Program at the University of Toronto

Javidan Arshia University of Toronto

Yang Samantha University of Toronto

Sutherland Travis University of Toronto

Li Yujin University of Toronto

Leo Joanne University of Toronto

Kulman-Lipsey Shayna University of Toronto

Perret Nellie University of Toronto

Trevelyan Christopher University of Toronto

Nickell Leslie University of Toronto

Rojas David University of Toronto

Electronic publication date: 2019 Apr 3

JOURNAL INFORMATION
==============================
NLM Title Abbreviation: Can Med Educ J
Journal ID: Can Med Educ J
NLM Journal ID: 101560935
Journal ID: CMEJ

Canadian Medical Education Journal

EISSN: 1923-1202
Publisher: University of Saskatchewan

ARTICLE INFORMATION
==============================
Subjects: Od - 8 – 4

Motivation in Medical School: A Quantitative Report on How the Learning Environment Influences Student Well-being and Resilience

Neufeld Adam University of Saskatchewan

Malin Greg University of Saskatchewan

McKay Shari University of Saskatchewan

Electronic publication date: 2019 Apr 3

JOURNAL INFORMATION
==============================
NLM Title Abbreviation: Can Med Educ J
Journal ID: Can Med Educ J
NLM Journal ID: 101560935
Journal ID: CMEJ

Canadian Medical Education Journal

EISSN: 1923-1202
Publisher: University of Saskatchewan

ARTICLE INFORMATION
==============================
Subjects: Od - 8 – 5

Understanding medical student perspectives of fatigue and fatigue risk management.

Raynard Alex Western University

Taylor Taryn Western University

Lingard Lorelei Western University

Electronic publication date: 2019 Apr 3

JOURNAL INFORMATION
==============================
NLM Title Abbreviation: Can Med Educ J
Journal ID: Can Med Educ J
NLM Journal ID: 101560935
Journal ID: CMEJ

Canadian Medical Education Journal

EISSN: 1923-1202
Publisher: University of Saskatchewan

ARTICLE INFORMATION
==============================
Subjects: Od - 8 – 6

Harassment and Bullying: Deleterious Impacts on Resident Wellness, Training and Performance

Maggi Julie University of Toronto

Babcock Glenys University of Toronto

Flett Heather University of Toronto

Ruetalo Mariela University of Toronto

Balakrishna Anita University of Toronto

Robinson Lisa University of Toronto

Bandiera Glen University of Toronto

Electronic publication date: 2019 Apr 3

JOURNAL INFORMATION
==============================
NLM Title Abbreviation: Can Med Educ J
Journal ID: Can Med Educ J
NLM Journal ID: 101560935
Journal ID: CMEJ

Canadian Medical Education Journal

EISSN: 1923-1202
Publisher: University of Saskatchewan

ARTICLE INFORMATION
==============================
Subjects: Oe - 1 – 1

Some run to them, others run away: how residents navigate learning of invasive procedures in the ICU setting

Brydges Ryan University of Toronto

Tran Judy University of Toronto

Lee Christie University of Toronto

Goffi Alberto University of Toronto

Mylopoulos Maria University of Toronto

Electronic publication date: 2019 Apr 3

JOURNAL INFORMATION
==============================
NLM Title Abbreviation: Can Med Educ J
Journal ID: Can Med Educ J
NLM Journal ID: 101560935
Journal ID: CMEJ

Canadian Medical Education Journal

EISSN: 1923-1202
Publisher: University of Saskatchewan

ARTICLE INFORMATION
==============================
Subjects: Oe - 1 – 2

Conversion to a customized learning platform enables active, formative learning in large size classrooms: student perceptions of the impact of online assessment using their own devices

Sibbald Debra University of Toronto

Electronic publication date: 2019 Apr 3

JOURNAL INFORMATION
==============================
NLM Title Abbreviation: Can Med Educ J
Journal ID: Can Med Educ J
NLM Journal ID: 101560935
Journal ID: CMEJ

Canadian Medical Education Journal

EISSN: 1923-1202
Publisher: University of Saskatchewan

ARTICLE INFORMATION
==============================
Subjects: Oe - 1 – 3

Square Pegs in Round Holes: Implementing Independent Learning in Structured Curricula

Rachul Christen University of Manitoba

Collins Benjamin University of Manitoba

Ahmed Mariam University of Manitoba

Cai George University of Manitoba

Electronic publication date: 2019 Apr 3

JOURNAL INFORMATION
==============================
NLM Title Abbreviation: Can Med Educ J
Journal ID: Can Med Educ J
NLM Journal ID: 101560935
Journal ID: CMEJ

Canadian Medical Education Journal

EISSN: 1923-1202
Publisher: University of Saskatchewan

ARTICLE INFORMATION
==============================
Subjects: Oe - 1 – 4

Converting the Academic Half Day to a Flipped Classroom Format - A Pilot Study of its Impact on Learner Behavioural Engagement

Nathoo Natasha McGill

Gomez-Garibello Carlos McGill

McGill Ning-Zi Sun

Electronic publication date: 2019 Apr 3

JOURNAL INFORMATION
==============================
NLM Title Abbreviation: Can Med Educ J
Journal ID: Can Med Educ J
NLM Journal ID: 101560935
Journal ID: CMEJ

Canadian Medical Education Journal

EISSN: 1923-1202
Publisher: University of Saskatchewan

ARTICLE INFORMATION
==============================
Subjects: Oe - 1 – 5

PlayMed: A randomised controlled trial for serious gaming in medical education

E. Perron Janaya University of New South Wales

Uther Penelope University of New South Wales

J. Coffey Michael University of New South Wales

Lovell-Simons Andrew University of New South Wales

Bartlett Adam University of New South Wales

M. McKay Ashlene University of New South Wales

Taylor Silas University of New South Wales

Garg Millie University of New South Wales

Lucas Sarah University of New South Wales

Cichero Jane University of New South Wales

Kennedy Sean University of New South Wales

Y. Ooi Chee University of New South Wales

Electronic publication date: 2019 Apr 3

JOURNAL INFORMATION
==============================
NLM Title Abbreviation: Can Med Educ J
Journal ID: Can Med Educ J
NLM Journal ID: 101560935
Journal ID: CMEJ

Canadian Medical Education Journal

EISSN: 1923-1202
Publisher: University of Saskatchewan

ARTICLE INFORMATION
==============================
Subjects: Oe - 1 – 6

Implementation of Native Apps for Logging Patient Encounters and Facilitating and Tracking Direct Observation and Feedback in Clerkship

Levinson Anthony McMaster University

Rudkowski Jill McMaster University

Menezes Natasja McMaster University

Baird Judy McMaster University

Whyte Rob McMaster University

Electronic publication date: 2019 Apr 3

JOURNAL INFORMATION
==============================
NLM Title Abbreviation: Can Med Educ J
Journal ID: Can Med Educ J
NLM Journal ID: 101560935
Journal ID: CMEJ

Canadian Medical Education Journal

EISSN: 1923-1202
Publisher: University of Saskatchewan

ARTICLE INFORMATION
==============================
Subjects: Oe - 2 – 1

Motivation to Access Laparoscopic Skills Training by Obstetrics and Gynaecology Residents: A Novel Tool to Characterize Motivation

Stairs Jocelyn Dalhousie University

W Bergey Bradley Queen's College, City University of New York

Scott Stephanie Dalhousie University

Electronic publication date: 2019 Apr 3

JOURNAL INFORMATION
==============================
NLM Title Abbreviation: Can Med Educ J
Journal ID: Can Med Educ J
NLM Journal ID: 101560935
Journal ID: CMEJ

Canadian Medical Education Journal

EISSN: 1923-1202
Publisher: University of Saskatchewan

ARTICLE INFORMATION
==============================
Subjects: Oe - 2 – 2

Improving Integrated Mental Health Care Rotations for Psychiatry Residents: Showing Incremental Evaluation and Change Works

Snelgrove Natasha University of Toronto

Levinson Andrea University of Toronto

Sunderji Nadiya University of Toronto

Electronic publication date: 2019 Apr 3

JOURNAL INFORMATION
==============================
NLM Title Abbreviation: Can Med Educ J
Journal ID: Can Med Educ J
NLM Journal ID: 101560935
Journal ID: CMEJ

Canadian Medical Education Journal

EISSN: 1923-1202
Publisher: University of Saskatchewan

ARTICLE INFORMATION
==============================
Subjects: Oe - 2 – 3

An examination of trends in patient-centered care, specific to the treatment of substance misuse and mental health conditions, using data from a family medicine residency program

Chapman Emily University of Alberta

Hamza Deena University of Alberta

Ross Shelley University of Alberta

Electronic publication date: 2019 Apr 3

JOURNAL INFORMATION
==============================
NLM Title Abbreviation: Can Med Educ J
Journal ID: Can Med Educ J
NLM Journal ID: 101560935
Journal ID: CMEJ

Canadian Medical Education Journal

EISSN: 1923-1202
Publisher: University of Saskatchewan

ARTICLE INFORMATION
==============================
Subjects: Oe - 2 – 4

Medical student perspectives on the impact of resident-as-teacher training: A realist review

Zondervan Nathan University of Calgary

McLaughlin Kevin University of Calgary

Electronic publication date: 2019 Apr 3

JOURNAL INFORMATION
==============================
NLM Title Abbreviation: Can Med Educ J
Journal ID: Can Med Educ J
NLM Journal ID: 101560935
Journal ID: CMEJ

Canadian Medical Education Journal

EISSN: 1923-1202
Publisher: University of Saskatchewan

ARTICLE INFORMATION
==============================
Subjects: Oe - 2 – 5

Uncovering the beginnings of Canadian anesthesiology to inform current residency training

Sharma Richa University of Toronto

Whitehead Cynthia University of Toronto

Kuper Ayelet University of Toronto

Electronic publication date: 2019 Apr 3

JOURNAL INFORMATION
==============================
NLM Title Abbreviation: Can Med Educ J
Journal ID: Can Med Educ J
NLM Journal ID: 101560935
Journal ID: CMEJ

Canadian Medical Education Journal

EISSN: 1923-1202
Publisher: University of Saskatchewan

ARTICLE INFORMATION
==============================
Subjects: Oe - 2 – 6

The Impact of Rural Rotations on Urban Based Postgraduate Learners

Ornstein Jodie University of Calgary

Malhi Rebecca University of Calgary

Myhre Douglas University of Calgary

Electronic publication date: 2019 Apr 3

JOURNAL INFORMATION
==============================
NLM Title Abbreviation: Can Med Educ J
Journal ID: Can Med Educ J
NLM Journal ID: 101560935
Journal ID: CMEJ

Canadian Medical Education Journal

EISSN: 1923-1202
Publisher: University of Saskatchewan

ARTICLE INFORMATION
==============================
Subjects: Oe - 3 – 1

Faculty Development in Competency-Based Medical Education: A Scoping Review of the Literature

Sirianni Giovanna University of Toronto

Glover-Takahashi Susan University of Toronto

Myers Jeffrey University of Toronto

Electronic publication date: 2019 Apr 3

JOURNAL INFORMATION
==============================
NLM Title Abbreviation: Can Med Educ J
Journal ID: Can Med Educ J
NLM Journal ID: 101560935
Journal ID: CMEJ

Canadian Medical Education Journal

EISSN: 1923-1202
Publisher: University of Saskatchewan

ARTICLE INFORMATION
==============================
Subjects: Oe - 3 – 2

Learner handover: does providing assessors with information about learners' weaknesses influence assessment scores?

Dory Valérie Université catholique de Louvain

Young Meredith McGill

Danoff Deborah McGill

Plotnick Laurie McGill

Pal Nicole McGill

Gumuchian Stephanie McGill

Dory Valerie McGill

Cummings Beth-Ann McGill

Gomez-Garibello Carlos McGill

Electronic publication date: 2019 Apr 3

JOURNAL INFORMATION
==============================
NLM Title Abbreviation: Can Med Educ J
Journal ID: Can Med Educ J
NLM Journal ID: 101560935
Journal ID: CMEJ

Canadian Medical Education Journal

EISSN: 1923-1202
Publisher: University of Saskatchewan

ARTICLE INFORMATION
==============================
Subjects: Oe - 3 – 3

Challenges and Solutions to the Implementation of CBME at Queens University

Chakraborty Amar Queen’s University

Tai Julia Queen’s University

Electronic publication date: 2019 Apr 3

JOURNAL INFORMATION
==============================
NLM Title Abbreviation: Can Med Educ J
Journal ID: Can Med Educ J
NLM Journal ID: 101560935
Journal ID: CMEJ

Canadian Medical Education Journal

EISSN: 1923-1202
Publisher: University of Saskatchewan

ARTICLE INFORMATION
==============================
Subjects: Oe - 3 – 5

The role of context in competence committee decision making

Acai Anita McMaster University

R. Sonnadara Ranil McMaster University

Electronic publication date: 2019 Apr 3

JOURNAL INFORMATION
==============================
NLM Title Abbreviation: Can Med Educ J
Journal ID: Can Med Educ J
NLM Journal ID: 101560935
Journal ID: CMEJ

Canadian Medical Education Journal

EISSN: 1923-1202
Publisher: University of Saskatchewan

ARTICLE INFORMATION
==============================
Subjects: Oe - 3 – 6

The effect of audio monitoring of oncology clinic medical students on the quality of consultants' written feedback

Sanatani Michael Western University

Potvin Kylea Western University

Conter Henry Western University

Trudgeon Kimberly Western University

Electronic publication date: 2019 Apr 3

JOURNAL INFORMATION
==============================
NLM Title Abbreviation: Can Med Educ J
Journal ID: Can Med Educ J
NLM Journal ID: 101560935
Journal ID: CMEJ

Canadian Medical Education Journal

EISSN: 1923-1202
Publisher: University of Saskatchewan

ARTICLE INFORMATION
==============================
Subjects: Oe - 4 – 1

Gendered Discourses about Parenthood in Canadian Medical Associations' Tax Advocacy

Cavanagh Alice McMaster University

Electronic publication date: 2019 Apr 3

JOURNAL INFORMATION
==============================
NLM Title Abbreviation: Can Med Educ J
Journal ID: Can Med Educ J
NLM Journal ID: 101560935
Journal ID: CMEJ

Canadian Medical Education Journal

EISSN: 1923-1202
Publisher: University of Saskatchewan

ARTICLE INFORMATION
==============================
Subjects: Oe - 4 – 2

Realist Synthesis of Educational Interventions Regarding Paediatric Pain

Sukhera Javeed Western University

Patterson Kyna Western University

Bertram Kaitlyn Western University

Electronic publication date: 2019 Apr 3

JOURNAL INFORMATION
==============================
NLM Title Abbreviation: Can Med Educ J
Journal ID: Can Med Educ J
NLM Journal ID: 101560935
Journal ID: CMEJ

Canadian Medical Education Journal

EISSN: 1923-1202
Publisher: University of Saskatchewan

ARTICLE INFORMATION
==============================
Subjects: Oe - 4 – 3

Why You Should Mini-Med School: Mini Med School as an Intervention to Increase Health Literacy

Harder Samuel University of British Columbia

Shatenko Sergiy University of British Columbia

Gair Jane University of British Columbia

Harder Samuel University of British Columbia

Electronic publication date: 2019 Apr 3

JOURNAL INFORMATION
==============================
NLM Title Abbreviation: Can Med Educ J
Journal ID: Can Med Educ J
NLM Journal ID: 101560935
Journal ID: CMEJ

Canadian Medical Education Journal

EISSN: 1923-1202
Publisher: University of Saskatchewan

ARTICLE INFORMATION
==============================
Subjects: Oe - 4 – 4

A unifying Faculty-wide vision for education: Innovation through stakeholder engagement

Majnemer Annette McGill

Thomas Aliki McGill

Bhanji Farhan McGill

Emed Jessica McGill

Finkelstein Adam McGill

Hébert Terence McGill

Kafantaris Demetra McGill

Lachapelle Kevin McGill

Razack Saleem McGill

Steinert Yvonne McGill

Young Meredith McGill

Electronic publication date: 2019 Apr 3

JOURNAL INFORMATION
==============================
NLM Title Abbreviation: Can Med Educ J
Journal ID: Can Med Educ J
NLM Journal ID: 101560935
Journal ID: CMEJ

Canadian Medical Education Journal

EISSN: 1923-1202
Publisher: University of Saskatchewan

ARTICLE INFORMATION
==============================
Subjects: Oe - 4 – 5

Students' Perception on use of Smartphones in Histology Teaching

El Bialy Safaa University of Ottawa

Lian ALexander Pearson University of Ottawa

Pearson Alexander University of Ottawa

Electronic publication date: 2019 Apr 3

JOURNAL INFORMATION
==============================
NLM Title Abbreviation: Can Med Educ J
Journal ID: Can Med Educ J
NLM Journal ID: 101560935
Journal ID: CMEJ

Canadian Medical Education Journal

EISSN: 1923-1202
Publisher: University of Saskatchewan

ARTICLE INFORMATION
==============================
Subjects: Oe - 5 – 1

Immersion, isolation, and enculturation in clinical training: An autoethnographic lens

Perrella Andrew University of Toronto

Coret Alon University of Toronto

Regehr Glenn University of British Columbia

Farrell Laura University of British Columbia

Electronic publication date: 2019 Apr 3

JOURNAL INFORMATION
==============================
NLM Title Abbreviation: Can Med Educ J
Journal ID: Can Med Educ J
NLM Journal ID: 101560935
Journal ID: CMEJ

Canadian Medical Education Journal

EISSN: 1923-1202
Publisher: University of Saskatchewan

ARTICLE INFORMATION
==============================
Subjects: Oe - 5 – 3

Resident perceptions of professional identity formation in a competency-based medical education framework

Cupido Nathan McMaster University

KN Tran Cindy McMaster University

Zering Jennifer McMaster University

R. Sonnadara Ranil McMaster University

Electronic publication date: 2019 Apr 3

JOURNAL INFORMATION
==============================
NLM Title Abbreviation: Can Med Educ J
Journal ID: Can Med Educ J
NLM Journal ID: 101560935
Journal ID: CMEJ

Canadian Medical Education Journal

EISSN: 1923-1202
Publisher: University of Saskatchewan

ARTICLE INFORMATION
==============================
Subjects: Oe - 5 – 4

Identity formation processes of Healthcare Students and Clinicians with Disabilities in their Educational Journeys

Mayer Yael University of British Columbia

Tal Jarus University of British Columbia

Shalev Michal University of British Columbia

Battalova Alfiya University of British Columbia

Yvonne Bulk Laura University of British Columbia

Gurdeep Parhar University of British Columbia

Lee Michael University of British Columbia

Nimmon Laura University of British Columbia

Electronic publication date: 2019 Apr 3

JOURNAL INFORMATION
==============================
NLM Title Abbreviation: Can Med Educ J
Journal ID: Can Med Educ J
NLM Journal ID: 101560935
Journal ID: CMEJ

Canadian Medical Education Journal

EISSN: 1923-1202
Publisher: University of Saskatchewan

ARTICLE INFORMATION
==============================
Subjects: Oe - 5 – 5

Lessons learned from a small group, inter-generational, narrative medicine program

Matos Meghan University of British Columbia

Yoo Jaeyun University of British Columbia

Bota Melissa University of British Columbia

Shklanka Karen University of British Columbia

Schrewe Brett University of British Columbia

Armstrong Linlea University of British Columbia

Electronic publication date: 2019 Apr 3

JOURNAL INFORMATION
==============================
NLM Title Abbreviation: Can Med Educ J
Journal ID: Can Med Educ J
NLM Journal ID: 101560935
Journal ID: CMEJ

Canadian Medical Education Journal

EISSN: 1923-1202
Publisher: University of Saskatchewan

ARTICLE INFORMATION
==============================
Subjects: Oe - 5 – 6

Remediating failure in medical students - using discourse analysis to understand the impacts on professional identity.

Read James University of Plymouth

Electronic publication date: 2019 Apr 3

JOURNAL INFORMATION
==============================
NLM Title Abbreviation: Can Med Educ J
Journal ID: Can Med Educ J
NLM Journal ID: 101560935
Journal ID: CMEJ

Canadian Medical Education Journal

EISSN: 1923-1202
Publisher: University of Saskatchewan

ARTICLE INFORMATION
==============================
Subjects: Oe - 6 – 1

Professionalism remediation: the relationship between severity of offence and quality of student insight as a function of the type of offence in McMaster's Undergraduate MD Program

Mountjoy Margo McMaster University

C. Burgess Raquel McMaster University

Vanstone Meredith McMaster University

E. M. Grierson Lawrence McMaster University

C. Burgess Raquel McMaster University

Electronic publication date: 2019 Apr 3

JOURNAL INFORMATION
==============================
NLM Title Abbreviation: Can Med Educ J
Journal ID: Can Med Educ J
NLM Journal ID: 101560935
Journal ID: CMEJ

Canadian Medical Education Journal

EISSN: 1923-1202
Publisher: University of Saskatchewan

ARTICLE INFORMATION
==============================
Subjects: Oe - 6 – 2

Conceptualizations of Professionalism: Investigating professionalism feedback in pre-clerkship medical education at the University of Toronto

Abner Erika University of Toronto

Rojas David University of Toronto

Tonin Paul University of Toronto

Nyhof-Young Joyce University of Toronto

Electronic publication date: 2019 Apr 3

JOURNAL INFORMATION
==============================
NLM Title Abbreviation: Can Med Educ J
Journal ID: Can Med Educ J
NLM Journal ID: 101560935
Journal ID: CMEJ

Canadian Medical Education Journal

EISSN: 1923-1202
Publisher: University of Saskatchewan

ARTICLE INFORMATION
==============================
Subjects: Oe - 6 – 3

Development of 'patient ownership' during clerkship

Leblanc Andréanne McGill

Snell Linda McGill

Sun Ning-Zi McGill

Electronic publication date: 2019 Apr 3

JOURNAL INFORMATION
==============================
NLM Title Abbreviation: Can Med Educ J
Journal ID: Can Med Educ J
NLM Journal ID: 101560935
Journal ID: CMEJ

Canadian Medical Education Journal

EISSN: 1923-1202
Publisher: University of Saskatchewan

ARTICLE INFORMATION
==============================
Subjects: Oe - 6 – 4

Exploring student perceptions when learning about patient partnerships with patient educators

Langlois Sylvia University of Toronto

Mehra Kamna Centre for Addiction and Mental Health

Electronic publication date: 2019 Apr 3

JOURNAL INFORMATION
==============================
NLM Title Abbreviation: Can Med Educ J
Journal ID: Can Med Educ J
NLM Journal ID: 101560935
Journal ID: CMEJ

Canadian Medical Education Journal

EISSN: 1923-1202
Publisher: University of Saskatchewan

ARTICLE INFORMATION
==============================
Subjects: Oe - 6 – 5

Examining how professional boundaries are shaped, perceived and maintained among family medicine residents and physicians

Bergin Fiona Dalhousie University

Alexiadis Brown Peggy Dalhousie University

Kyte Darrell Dalhousie University

Electronic publication date: 2019 Apr 3

JOURNAL INFORMATION
==============================
NLM Title Abbreviation: Can Med Educ J
Journal ID: Can Med Educ J
NLM Journal ID: 101560935
Journal ID: CMEJ

Canadian Medical Education Journal

EISSN: 1923-1202
Publisher: University of Saskatchewan

ARTICLE INFORMATION
==============================
Subjects: Oe - 6 – 6

Are we trying to teach the unteachable? Beliefs held by clinical supervisors towards professional attributes

Pal Nicole McGill

Meredith Young Dr. McGill

Laurie Plotnick Dr. McGill

Deborah Danoff Dr. McGill

Beth-Ann Cummings Dr. McGill

Carlos Gomez-Garibello Dr. McGill

Valérie Dory Dr. McGill

Electronic publication date: 2019 Apr 3

JOURNAL INFORMATION
==============================
NLM Title Abbreviation: Can Med Educ J
Journal ID: Can Med Educ J
NLM Journal ID: 101560935
Journal ID: CMEJ

Canadian Medical Education Journal

EISSN: 1923-1202
Publisher: University of Saskatchewan

ARTICLE INFORMATION
==============================
Subjects: Of - 1 – 1

Using learning curves to identify and explain growth patterns of learners in bronchoscopy simulation: a mixed method study

Mema Briseida University of Toronto

Mylopoulos Maria University of Toronto

Soo Park Yoon University of Illinois

Electronic publication date: 2019 Apr 3

JOURNAL INFORMATION
==============================
NLM Title Abbreviation: Can Med Educ J
Journal ID: Can Med Educ J
NLM Journal ID: 101560935
Journal ID: CMEJ

Canadian Medical Education Journal

EISSN: 1923-1202
Publisher: University of Saskatchewan

ARTICLE INFORMATION
==============================
Subjects: Of - 1 – 2

Enhancing medical learners' knowledge of, comfort and confidence in holding Goals-of-Care Conversations: an adaptation of the Serious Illness Care Program

Tam Vivian University of Toronto

You John McMaster University

Electronic publication date: 2019 Apr 3

JOURNAL INFORMATION
==============================
NLM Title Abbreviation: Can Med Educ J
Journal ID: Can Med Educ J
NLM Journal ID: 101560935
Journal ID: CMEJ

Canadian Medical Education Journal

EISSN: 1923-1202
Publisher: University of Saskatchewan

ARTICLE INFORMATION
==============================
Subjects: Of - 1 – 3

The impact of clinical environments on trust-building between medical student clerks and their teachers

Block Emily McMaster University

Bell Amanda McMaster University

Walsh Allyn McMaster University

Stobbe Karl McMaster University

Vanstone Meredith McMaster University

Electronic publication date: 2019 Apr 3

JOURNAL INFORMATION
==============================
NLM Title Abbreviation: Can Med Educ J
Journal ID: Can Med Educ J
NLM Journal ID: 101560935
Journal ID: CMEJ

Canadian Medical Education Journal

EISSN: 1923-1202
Publisher: University of Saskatchewan

ARTICLE INFORMATION
==============================
Subjects: Of - 1 – 4

Evaluating the impact of the Annual Refresher Courses for Family Physicians on reported practice outcomes

Francesca Luconi Dr. McGill

Ivan Rohan Dr. McGill

Meron Teferra Ms. McGill

Inas Malaty Ms. McGill

Electronic publication date: 2019 Apr 3

JOURNAL INFORMATION
==============================
NLM Title Abbreviation: Can Med Educ J
Journal ID: Can Med Educ J
NLM Journal ID: 101560935
Journal ID: CMEJ

Canadian Medical Education Journal

EISSN: 1923-1202
Publisher: University of Saskatchewan

ARTICLE INFORMATION
==============================
Subjects: Of - 1 – 5

Probeficiency: an innovative peer-led model for providing weekly free and open-access Point of Care Ultrasound (PoCUS) education

Mei Liu Xin University of Ottawa

Tran Brian McGill

Caminsky Natasha McGill

Electronic publication date: 2019 Apr 3

JOURNAL INFORMATION
==============================
NLM Title Abbreviation: Can Med Educ J
Journal ID: Can Med Educ J
NLM Journal ID: 101560935
Journal ID: CMEJ

Canadian Medical Education Journal

EISSN: 1923-1202
Publisher: University of Saskatchewan

ARTICLE INFORMATION
==============================
Subjects: Of - 1 – 6

What makes surgical boot camps effective? New insights using a mixed methods approach

Wagner Natalie McMaster University

Amin Nalin McMaster University

Kelly Stephen McMaster University

Sonnadara Ranil McMaster University

Electronic publication date: 2019 Apr 3

JOURNAL INFORMATION
==============================
NLM Title Abbreviation: Can Med Educ J
Journal ID: Can Med Educ J
NLM Journal ID: 101560935
Journal ID: CMEJ

Canadian Medical Education Journal

EISSN: 1923-1202
Publisher: University of Saskatchewan

ARTICLE INFORMATION
==============================
Subjects: Of - 2 – 1

How residents manage dual expectations to care and cure

Arya Rigya Western University

Thain Jenny Western University

Diachun Laura Western University

Cristancho Sayra Western University

Electronic publication date: 2019 Apr 3

JOURNAL INFORMATION
==============================
NLM Title Abbreviation: Can Med Educ J
Journal ID: Can Med Educ J
NLM Journal ID: 101560935
Journal ID: CMEJ

Canadian Medical Education Journal

EISSN: 1923-1202
Publisher: University of Saskatchewan

ARTICLE INFORMATION
==============================
Subjects: Of - 2 – 2

How do my elective choices relate to CaRMS Match success?

Lee Robert CaRMS

Bandiera Glen University of Toronto

Ouellette Michel CaRMS

Gallinger John CaRMS

Electronic publication date: 2019 Apr 3

JOURNAL INFORMATION
==============================
NLM Title Abbreviation: Can Med Educ J
Journal ID: Can Med Educ J
NLM Journal ID: 101560935
Journal ID: CMEJ

Canadian Medical Education Journal

EISSN: 1923-1202
Publisher: University of Saskatchewan

ARTICLE INFORMATION
==============================
Subjects: Of - 2 – 3

Getting to capability: how trainees adjust to new clinical workplace contexts

Watling Christopher Western University

Tessaro James University of British Columbia

Myers Kathy Western University

Bates Joanna University of British Columbia

Topps Maureen University of Calgary

Teunissen Pim University of Maastricht

Ellaway Rachel University of Calgary

Asgarova Sevinj University of British Columbia

Electronic publication date: 2019 Apr 3

JOURNAL INFORMATION
==============================
NLM Title Abbreviation: Can Med Educ J
Journal ID: Can Med Educ J
NLM Journal ID: 101560935
Journal ID: CMEJ

Canadian Medical Education Journal

EISSN: 1923-1202
Publisher: University of Saskatchewan

ARTICLE INFORMATION
==============================
Subjects: Of - 2 – 4

Assessment of implementing practice-based small group learning as part of academic half-days.

Armson Heather University of Calgary

Wycliffe-Jones Keith University of Calgary

Palacios Mone University of Calgary

Roder Stefanie The Foundation for Medical Practice Education

Electronic publication date: 2019 Apr 3

JOURNAL INFORMATION
==============================
NLM Title Abbreviation: Can Med Educ J
Journal ID: Can Med Educ J
NLM Journal ID: 101560935
Journal ID: CMEJ

Canadian Medical Education Journal

EISSN: 1923-1202
Publisher: University of Saskatchewan

ARTICLE INFORMATION
==============================
Subjects: Of - 2 – 5

"Shaking-Up" our Multidisciplinary Healthcare Learning and Working Environments - Resident Doctors of Canada's Toolkit for Positive Change

Bahji Anees Queen’s University

Amin Aditi University of Alberta

Lip Alyssa University of Calgary

Ahmad Tehmina University of Toronto

Electronic publication date: 2019 Apr 3

JOURNAL INFORMATION
==============================
NLM Title Abbreviation: Can Med Educ J
Journal ID: Can Med Educ J
NLM Journal ID: 101560935
Journal ID: CMEJ

Canadian Medical Education Journal

EISSN: 1923-1202
Publisher: University of Saskatchewan

ARTICLE INFORMATION
==============================
Subjects: Of - 2 – 6

Fundamental trends within falling match rates: novel insights from 10 years of CaRMS data

Zeng Andy University of Toronto

Brenna Connor University of Toronto

Ndoja Silvio Western University

Electronic publication date: 2019 Apr 3

JOURNAL INFORMATION
==============================
NLM Title Abbreviation: Can Med Educ J
Journal ID: Can Med Educ J
NLM Journal ID: 101560935
Journal ID: CMEJ

Canadian Medical Education Journal

EISSN: 1923-1202
Publisher: University of Saskatchewan

ARTICLE INFORMATION
==============================
Subjects: Of - 3 – 1

Remediation of Struggling Physicians

Armson Heather University of Calgary

Cooke Lara University of Calgary

Burak Kelly University of Calgary

Wickland-Weller Monica Monica.Wickland-Weller@cpsa.ab.ca

Electronic publication date: 2019 Apr 3

JOURNAL INFORMATION
==============================
NLM Title Abbreviation: Can Med Educ J
Journal ID: Can Med Educ J
NLM Journal ID: 101560935
Journal ID: CMEJ

Canadian Medical Education Journal

EISSN: 1923-1202
Publisher: University of Saskatchewan

ARTICLE INFORMATION
==============================
Subjects: Of - 3 – 2

Remediating doctors' performance to restore patient safety: A realist review

Price Tristan University of Plymouth

Archer Julian University of Plymouth

Cleland Jennifer University of Aberdeen

Prescott-Clements Linda Royal College of Veterinary Surgeons

Wanner Amanda University of Plymouth

Withers Lyndsey University of Plymouth

Wong Geoff University of Oxford

Brennan Nicola University of Plymouth

Electronic publication date: 2019 Apr 3

JOURNAL INFORMATION
==============================
NLM Title Abbreviation: Can Med Educ J
Journal ID: Can Med Educ J
NLM Journal ID: 101560935
Journal ID: CMEJ

Canadian Medical Education Journal

EISSN: 1923-1202
Publisher: University of Saskatchewan

ARTICLE INFORMATION
==============================
Subjects: Of - 3 – 3

Improving Self-Regulation of Learning Amongst Underperforming Medical Students: An Embedded Mixed Methods Study

Desy Janeve University of Calgary

McLaughlin Kevin University of Calgary

Coderre Sylvain University of Calgary

Bryant Camille Johns Hopkins University

Veale Pamela University of Calgary

Busche Kevin University of Calgary

Kachra Rahim University of Calgary

Bass Adam University of Calgary

Ruzycki Shannon University of Calgary

Ranelli Luke University of Calgary

Paget Mike University of Calgary

Sobczak Mateusz University of Calgary

Electronic publication date: 2019 Apr 3

JOURNAL INFORMATION
==============================
NLM Title Abbreviation: Can Med Educ J
Journal ID: Can Med Educ J
NLM Journal ID: 101560935
Journal ID: CMEJ

Canadian Medical Education Journal

EISSN: 1923-1202
Publisher: University of Saskatchewan

ARTICLE INFORMATION
==============================
Subjects: Of - 3 – 4

An Environmental Scan of Canadian Physician Re-Entry, Remediation, & Re-Training Programs

Fleet Lisa Memorial – University of Newfoundland

Curran Vernon Memorial – University of Newfoundland

Electronic publication date: 2019 Apr 3

JOURNAL INFORMATION
==============================
NLM Title Abbreviation: Can Med Educ J
Journal ID: Can Med Educ J
NLM Journal ID: 101560935
Journal ID: CMEJ

Canadian Medical Education Journal

EISSN: 1923-1202
Publisher: University of Saskatchewan

ARTICLE INFORMATION
==============================
Subjects: Of - 3 – 5

Community-based rotations and professional identity development

Watling Christopher Western University

Bates Joanna University of British Columbia

Teunissen Pim University of Maastricht

Topps Maureen University of Calgary

Ellaway Rachel University of Calgary

Myers Kathy Western University

Tessaro James University of British Columbia

Asgarova Sevinj University of British Columbia

Electronic publication date: 2019 Apr 3

JOURNAL INFORMATION
==============================
NLM Title Abbreviation: Can Med Educ J
Journal ID: Can Med Educ J
NLM Journal ID: 101560935
Journal ID: CMEJ

Canadian Medical Education Journal

EISSN: 1923-1202
Publisher: University of Saskatchewan

ARTICLE INFORMATION
==============================
Subjects: Of - 4 – 1

Research and Reconciliation: Exploring the approaches of non-Indigenous researchers to Indigenous research

Kilian Alexandra University of Toronto

Kuper Ayelet University of Toronto

Whitehead Cynthia University of Toronto

Richardson Lisa University of Toronto

Giroux Ryan University of Toronto

Fellows Tyee University of Toronto

Pennington Jason University of Toronto

Electronic publication date: 2019 Apr 3

JOURNAL INFORMATION
==============================
NLM Title Abbreviation: Can Med Educ J
Journal ID: Can Med Educ J
NLM Journal ID: 101560935
Journal ID: CMEJ

Canadian Medical Education Journal

EISSN: 1923-1202
Publisher: University of Saskatchewan

ARTICLE INFORMATION
==============================
Subjects: Of - 4 – 2

Curriculum mapping, Systems Engineering, and Program Evaluation: Determining the impact of the MD program curriculum at the University of Toronto.

Rojas David University of Toronto

Electronic publication date: 2019 Apr 3

JOURNAL INFORMATION
==============================
NLM Title Abbreviation: Can Med Educ J
Journal ID: Can Med Educ J
NLM Journal ID: 101560935
Journal ID: CMEJ

Canadian Medical Education Journal

EISSN: 1923-1202
Publisher: University of Saskatchewan

ARTICLE INFORMATION
==============================
Subjects: Of - 4 – 3

Utilizing student assessment data analytics for post-renewal analysis and future planning in the University of Toronto MD Program

Howard Frazer University of Toronto

Pittini Richard University of Toronto

Tait Glendon University of Toronto

Kulasegaram Kulamakan University of Toronto

Charles Tamica University of Toronto

Pan Pauline University of Toronto

Rojas David University of Toronto

Electronic publication date: 2019 Apr 3

JOURNAL INFORMATION
==============================
NLM Title Abbreviation: Can Med Educ J
Journal ID: Can Med Educ J
NLM Journal ID: 101560935
Journal ID: CMEJ

Canadian Medical Education Journal

EISSN: 1923-1202
Publisher: University of Saskatchewan

ARTICLE INFORMATION
==============================
Subjects: Of - 4 – 4

Continuing professional development and organizational logics: Participating in the governing of health care professionals in workplaces

Rowland Paula University of Toronto

Boyd Victoria University of Toronto

Goldman Joanne University of Toronto

Lising Dean University of Toronto

Ng Stella University of Toronto

Whitehead Cynthia University of Toronto

Electronic publication date: 2019 Apr 3

JOURNAL INFORMATION
==============================
NLM Title Abbreviation: Can Med Educ J
Journal ID: Can Med Educ J
NLM Journal ID: 101560935
Journal ID: CMEJ

Canadian Medical Education Journal

EISSN: 1923-1202
Publisher: University of Saskatchewan

ARTICLE INFORMATION
==============================
Subjects: Of - 4 – 5

Exploring the ethics of authorship in academic medicine

R. Baker Lindsay University of Toronto

Friesen Farah University of Toronto

L. Ng Stella University of Toronto

Camp Mark SickKids

Szego Michael St. Michael's Hospital

Electronic publication date: 2019 Apr 3

JOURNAL INFORMATION
==============================
NLM Title Abbreviation: Can Med Educ J
Journal ID: Can Med Educ J
NLM Journal ID: 101560935
Journal ID: CMEJ

Canadian Medical Education Journal

EISSN: 1923-1202
Publisher: University of Saskatchewan

ARTICLE INFORMATION
==============================
Subjects: Of - 4 – 6

Identifying and communicating actual value of our work via a Theory of Impact: Stories from the field of education scholarship

Parker Kathryn University of Toronto

Cartmill Carrie University of Toronto

Ghavam-Rassoul Abbas University of Toronto

Nutik Melissa University of Toronto

Tannenbaum David University of Toronto

Wright Sarah University of Toronto

Woods Nicole University of Toronto

Freeman Risa University of Toronto

Electronic publication date: 2019 Apr 3

JOURNAL INFORMATION
==============================
NLM Title Abbreviation: Can Med Educ J
Journal ID: Can Med Educ J
NLM Journal ID: 101560935
Journal ID: CMEJ

Canadian Medical Education Journal

EISSN: 1923-1202
Publisher: University of Saskatchewan

ARTICLE INFORMATION
==============================
Subjects: Of - 5 – 1

The Role of Simulation-Based Education in Teaching Outside of Operating Room Difficult Airway Management

Beecroft James Niagara Health

Chirico John Niagara Health

Shira Brown N. Niagara Health

Electronic publication date: 2019 Apr 3

JOURNAL INFORMATION
==============================
NLM Title Abbreviation: Can Med Educ J
Journal ID: Can Med Educ J
NLM Journal ID: 101560935
Journal ID: CMEJ

Canadian Medical Education Journal

EISSN: 1923-1202
Publisher: University of Saskatchewan

ARTICLE INFORMATION
==============================
Subjects: Of - 5 – 2

Learning after the simulation is done: The role of simulation in supporting ongoing self-regulated learning

Shariff Farhana University of British Columbia

Regehr Glenn University of British Columbia

Hatala Rose University of British Columbia

Electronic publication date: 2019 Apr 3

JOURNAL INFORMATION
==============================
NLM Title Abbreviation: Can Med Educ J
Journal ID: Can Med Educ J
NLM Journal ID: 101560935
Journal ID: CMEJ

Canadian Medical Education Journal

EISSN: 1923-1202
Publisher: University of Saskatchewan

ARTICLE INFORMATION
==============================
Subjects: Of - 5 – 3

Identifying Resuscitation Expertise Using Cognitive Load: Evidence from a Pulmonary Embolism Simulation Exercise

Bruder Eric Queen’s University

Dalgarno Nancy Queen’s University

Cofie Nicholas Queen’s University

Belyea Andrew Queen’s University

Electronic publication date: 2019 Apr 3

JOURNAL INFORMATION
==============================
NLM Title Abbreviation: Can Med Educ J
Journal ID: Can Med Educ J
NLM Journal ID: 101560935
Journal ID: CMEJ

Canadian Medical Education Journal

EISSN: 1923-1202
Publisher: University of Saskatchewan

ARTICLE INFORMATION
==============================
Subjects: Of - 5 – 4

A qualitative study of epistemologies in a simulation-based medical education context

Ng Stella University of Toronto

Brydges Ryan University of Toronto

Kangasjarvi Emilia St. Michael's Hospital

Lorello Gianni University of Toronto

Nemoy Lori St. Michael's Hospital

Electronic publication date: 2019 Apr 3

JOURNAL INFORMATION
==============================
NLM Title Abbreviation: Can Med Educ J
Journal ID: Can Med Educ J
NLM Journal ID: 101560935
Journal ID: CMEJ

Canadian Medical Education Journal

EISSN: 1923-1202
Publisher: University of Saskatchewan

ARTICLE INFORMATION
==============================
Subjects: Of - 5 – 5

"Limbs and things": A sociomaterial ethnography of undergraduate medical simulation using mannequins

Cameron Paula Dalhousie University

MacLeod Anna Dalhousie University

Kits Olga Dalhousie University

Tummons Jonathan Durham University

Cleveland-Innes Martha Athabasca University

Ajjawi Rola Deakin University

Electronic publication date: 2019 Apr 3

JOURNAL INFORMATION
==============================
NLM Title Abbreviation: Can Med Educ J
Journal ID: Can Med Educ J
NLM Journal ID: 101560935
Journal ID: CMEJ

Canadian Medical Education Journal

EISSN: 1923-1202
Publisher: University of Saskatchewan

ARTICLE INFORMATION
==============================
Subjects: Of - 5 – 6

Multi-Patient Simulation with Standardized Patients in Undergraduate Medical Education

Seto Anthony University of Calgary

Crooks Sean University of Calgary

Streith Lucas University of Calgary

Electronic publication date: 2019 Apr 3

JOURNAL INFORMATION
==============================
NLM Title Abbreviation: Can Med Educ J
Journal ID: Can Med Educ J
NLM Journal ID: 101560935
Journal ID: CMEJ

Canadian Medical Education Journal

EISSN: 1923-1202
Publisher: University of Saskatchewan

ARTICLE INFORMATION
==============================
Subjects: Of - 6 – 1

Modeling Ethics and Empathy in Medical Education

Platt Elyse McMaster University

Holland Alyson McMaster University

Electronic publication date: 2019 Apr 3

JOURNAL INFORMATION
==============================
NLM Title Abbreviation: Can Med Educ J
Journal ID: Can Med Educ J
NLM Journal ID: 101560935
Journal ID: CMEJ

Canadian Medical Education Journal

EISSN: 1923-1202
Publisher: University of Saskatchewan

ARTICLE INFORMATION
==============================
Subjects: Of - 6 – 3

Gender effects in assessment of clinical teaching: Does concordance matter?

Stroud Lynfa University of Toronto

Freeman Risa University of Toronto

Kulasegaram Mahan University of Toronto, Tulin Cil University of Toronto

Ginsburg Shiphra University of Toronto

Electronic publication date: 2019 Apr 3

JOURNAL INFORMATION
==============================
NLM Title Abbreviation: Can Med Educ J
Journal ID: Can Med Educ J
NLM Journal ID: 101560935
Journal ID: CMEJ

Canadian Medical Education Journal

EISSN: 1923-1202
Publisher: University of Saskatchewan

ARTICLE INFORMATION
==============================
Subjects: Of - 6 – 4

Teacher Self-Efficacy (TSE) of Recently Graduated Emergency Medicine Physicians and the Factors Influencing TSE

Al Khamisi Aisha McGill

Meyers Chrisitine McGill

Petrosoniak Andrew University of Toronto

Ruglis Jessica McGill

Electronic publication date: 2019 Apr 3

JOURNAL INFORMATION
==============================
NLM Title Abbreviation: Can Med Educ J
Journal ID: Can Med Educ J
NLM Journal ID: 101560935
Journal ID: CMEJ

Canadian Medical Education Journal

EISSN: 1923-1202
Publisher: University of Saskatchewan

ARTICLE INFORMATION
==============================
Subjects: Of - 6 – 5

Conceptualizations, teaching practices and collaborations in the development of learners' EBP competencies: Faculty and clinical supervisors' perspectives on teaching EBP to rehabilitation students

Hallé Marie-Christine McGill

Thomas Aliki McGill

Bussières André McGill

Asseraf-Pasin Liliane McGill

Mak Susanne McGill

Root Kelly McGill

Steinhauer Karsten McGill

Storr Caroline McGill

Vaillancourt Sophie McGill

Electronic publication date: 2019 Apr 3

JOURNAL INFORMATION
==============================
NLM Title Abbreviation: Can Med Educ J
Journal ID: Can Med Educ J
NLM Journal ID: 101560935
Journal ID: CMEJ

Canadian Medical Education Journal

EISSN: 1923-1202
Publisher: University of Saskatchewan

ARTICLE INFORMATION
==============================
Subjects: Of - 6 – 6

Moving beyond training for process skills: Focusing in on what matters when exploring patient preferences around resuscitation

Lassaline Rachelle Western University

Taneja Ravi Western University

Goldszmidt Mark Western University

Torti Jacqueline Western University

Electronic publication date: 2019 Apr 3

